# Regenerative potential of epicardium-derived extracellular vesicles mediated by conserved miRNA transfer

**DOI:** 10.1093/cvr/cvab054

**Published:** 2021-02-18

**Authors:** Cristina Villa del Campo, Norman Y Liaw, Mala Gunadasa-Rohling, Moritz Matthaei, Luca Braga, Tahnee Kennedy, Gabriela Salinas, Niels Voigt, Mauro Giacca, Wolfram-Hubertus Zimmermann, Paul Richard Riley

**Affiliations:** 1 Department of Physiology, Anatomy and Genetics, British Heart Foundation, Oxbridge Centre of Regenerative Medicine, University of Oxford, Sherrington Building, Sherrington Rd, Oxford OX1 3PT, UK; 2 Institute of Pharmacology and Toxicology, University Medical Center Göttingen, Robert-Koch-Str. 40, 37075 Göttingen, Germany; 3 DZHK (German Centre for Cardiovascular Research), partner site Göttingen, Robert-Koch-Straße 42a, 37075 Göttingen, Germany; 4 Molecular Medicine Laboratory, International Centre for Genetic Engineering and Biotechnology (ICGEB), Località Padriciano, 99, 34149 Trieste TS, Italy; 5 School of Cardiovascular Medicine & Sciences, British Heart Foundation Centre, King's College London, Strand, London WC2R 2L, UK; 6 NGS- Integrative Genomics Core Unit (NIG), Institute of Human Genetics, University Medical Centre Göttingen (UMG), Robert-Koch-Str. 40, 37075 Göttingen, Germany

**Keywords:** Epicardium, Extracellular vesicles, Myocardial infarction, Regeneration, FUCCI, MicroRNA, Human engineered myocardium

## Abstract

**Aims:**

After a myocardial infarction, the adult human heart lacks sufficient regenerative capacity to restore lost tissue, leading to heart failure progression. Finding novel ways to reprogram adult cardiomyocytes into a regenerative state is a major therapeutic goal. The epicardium, the outermost layer of the heart, contributes cardiovascular cell types to the forming heart and is a source of trophic signals to promote heart muscle growth during embryonic development. The epicardium is also essential for heart regeneration in zebrafish and neonatal mice and can be reactivated after injury in adult hearts to improve outcome. A recently identified mechanism of cell–cell communication and signalling is that mediated by extracellular vesicles (EVs). Here, we aimed to investigate epicardial signalling *via* EV release in response to cardiac injury and as a means to optimize cardiac repair and regeneration.

**Methods and results:**

We isolated epicardial EVs from mouse and human sources and targeted the cardiomyocyte population. Epicardial EVs enhanced proliferation in H9C2 cells and in primary neonatal murine cardiomyocytes *in vitro* and promoted cell cycle re-entry when injected into the injured area of infarcted neonatal hearts. These EVs also enhanced regeneration in cryoinjured engineered human myocardium (EHM) as a novel model of human myocardial injury. Deep RNA-sequencing of epicardial EV cargo revealed conserved microRNAs (miRs) between human and mouse epicardial-derived exosomes, and the effects on cell cycle re-entry were recapitulated by administration of cargo miR-30a, miR-100, miR-27a, and miR-30e to human stem cell-derived cardiomyocytes and cryoinjured EHM constructs.

**Conclusion:**

Here, we describe the first characterization of epicardial EV secretion, which can signal to promote proliferation of cardiomyocytes in infarcted mouse hearts and in a human model of myocardial injury, resulting in enhanced contractile function. Analysis of exosome cargo in mouse and human identified conserved pro-regenerative miRs, which in combination recapitulated the therapeutic effects of promoting cardiomyocyte proliferation.

## Graphical Abstract

**Figure:**
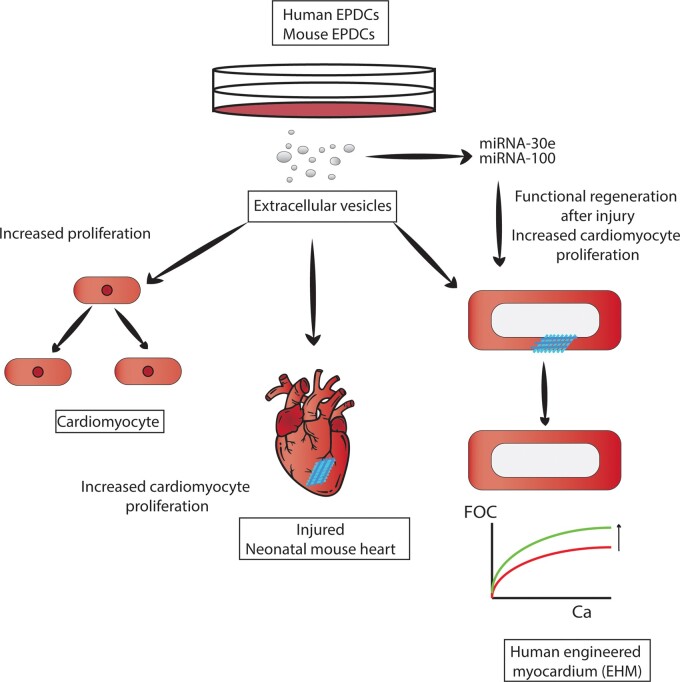

**Time for primary review: 30 days**

## 1. Introduction

Cardiovascular diseases (CVD) are one of the leading causes of death worldwide.^1^ Sublethal cardiac ischaemic events lead to the development of a non-contractile myocardium that evokes pathological compensatory mechanisms within the remaining viable tissue. If the damage cannot be compensated, progression towards heart failure ensues. Current state-of-the-art pharmacotherapies aim at protecting the heart from neurohumoral stressors and reducing the metabolic burden of the impaired myocardium. Replacement of the underlying loss of functional heart muscle *via* stimulating cardiomyocyte regeneration is not achieved with current treatments. Therefore, significant efforts have been made to develop alternate cell-based therapies, including those aimed at promoting cardiomyocyte proliferation to regenerate the injured heart and prevent the onset of heart failure.

Targeting the low proliferative capacity of adult human cardiomyocytes^2^ to re-enter the cell cycle is a major therapeutic goal. Several cell types with an innate capacity to provide regenerative cues have been identified, such as the epicardium.^3–5^ Epicardial cells form the outermost layer of the heart, and in adults, they are essentially quiescent with low-level turnover to maintain myocardial homeostasis. During cardiac development, the epicardium contributes key cell types, such as fibroblasts^6^ and vascular smooth muscle cells^6^ and endothelial cells to the developing coronaries.^7^^,^^8^ The epicardium also promotes cardiomyocyte proliferation *via* paracrine signalling.^9^^,^^10^ Furthermore, epicardial and epicardium-derived cells (EPDCs) reinvoke an embryonic gene programme and developmental characteristics after injury in lower vertebrates^11^^,^^12^ and regenerative murine models.^13^ This in turn can be induced in adult mammalian hearts after injury by priming with factors, such as the small peptide Thymosin β4 to improve outcome in the non-regenerative setting.^4^^,^^14^

As in development, a key aspect of the pro-regenerative function of the epicardium is via signalling and provision of mitogens and trophic growth factors. In zebrafish, retinoic acid production from the epicardium was shown to be essential for heart regeneration^15^ and in mouse epicardium, retinoid X receptor alpha is known to promote foetal cardiomyocyte proliferation.^9^ Whether epicardial signals can act to promote cardiomyocyte proliferation in adult mammalian hearts, either in steady state or following injury remains unknown.

Recently, there has been a significant focus around alternate mechanisms of intercellular signalling and novel modes of cell–cell communication mediated by extracellular vesicles (EVs) and the subclass known as exosomes.^16^ EVs were first identified as a subpopulation of double-membrane vesicles that are secreted by cells in culture.^17^ They are endosomal in origin, express specific membrane markers^18^^,^^19^ and have a defined size, ranging from 30 to 120 nm. EVs and exosomes have been described as potential biomarkers of cellular damage in plasma^20^ and pericardial fluid,^21^ and have also been proposed as a novel mode to deliver therapeutic factors. They are vehicles for mitogenic cargo, possess low immunogenicity, can be cryopreserved and are able to be isolated in large quantities.^22^ EVs and exosomes derived from plasma,^23^ mesenchymal stem cells,^24–26^ embryonic stem cells,^27^ cardiosphere-derived cells,^28^ bone marrow,^29^ and cardiac progenitor cells^30^ have been reported to be cardioprotective and improve outcome following ischaemic heart injury.

In this study, we describe for the first time the secretion of mouse and human EVs by epicardial cells, and their ability to evoke a cardiomyocyte proliferative response in regenerative postnatal day 1 (P1) and non-regenerative P7 neonatal mice^13^ after myocardial infarction (MI), and in human embryonic stem (hES) cell-derived cardiomyocytes *in vitro*. We selected neonatal mouse heart injury to model cardiovascular regeneration vs. repair whereby we can assess the documented shift from regeneration underpinned by cardiomyocyte proliferation to an adult default wound healing process of fibrosis. Injured P7 hearts respond by scarring and remodelling after injury and are unable to induce a proliferative response in the cardiomyocyte population to restore lost myocardial tissue; in this regard they mimic the non-regenerative response observed in adult hearts.^13^^,^^31^^,^^32^ Employing the neonatal MI model enables us to determine effects of EVs on existing cardiomyocyte proliferative capacity at P1 vs. a direct comparison 7 days later when this proliferative capacity is negligible. We also utilized engineered human myocardium (EHM^33^) to test the effect of acute EV delivery after cryoinjury. EHM is a human model, which parallel the neonatal mouse system via a more developmental and potentially regenerative response to injury, allowing for comparative analyses following EV treatment across both models. We describe here the first demonstration of (cryo) injury response in EHM, which mimics cell and tissue loss, as observed in AMI patients, and as such facilitates testing of the potential therapeutic effects of EVs. Contractile recovery of EHM was restored within 7 days post-injury following treatment with EVs, *via* increased cell cycle re-entry of the remaining cardiomyocyte population. Analysis of microRNA cargo of mouse and human epicardial EVs revealed conserved miR content across species. Adenoviral-mediated delivery of highly represented miRNAs promoted proliferation of human cardiomyocytes recapitulating the mitogenic effects of host EVs. These findings are of widespread interest in terms of understanding cell-cell communication in the context of ischaemic heart disease and pave the way to develop new approaches to promote myocardial tissue restoration based on paracrine signalling mediated by epicardial EV release.

## 2. Methods

### 2.1 Institutional ethics

All surgical and pharmacological procedures were performed in accordance with the Animals (Scientific Procedures) Act 1986 (ASPA) (Home Office, UK). All animal experiments were subject to local ethical committee approval (ACER) and all were undertaken in compliance with the standards set down in the ASPA, revised 2012, and with the requirements of the European Directive 63/2010 on the protection of animals used for scientific purposes. Home Office project licences (PPL 30/2987; PPDE89C84). The use of human embryonic stem cells (hESCs) for the tissue engineering work was approved according to the German Stem Cell Act by the Robert-Koch-Institute (permit #12; reference number: 1710-79-1-4-16).

### 2.2 Animal husbandry, MI, and EV delivery in postnatal mice

Mice were housed and maintained in a controlled environment by University of Oxford Biomedical Services. FUCCI R26Rp-mice were kept on a C57BL/6J inbred background and have been described previously.^34^ Animals were euthanized by cervical dislocation.

Neonatal (P1/P7) mice used in MI experiments were anaesthetized using a combination of hypothermia and inhaled isoflurane 3% v/v in oxygen. Neonates were exposed to this for 15 s followed by ice for 30–45 s for a P1 pup or 60–90 s for a P7 pup. MI was performed by permanent ligation of the left anterior descending^35^ coronary artery. For sham controls, a suture was passed under the LAD but not ligated. Mice treated with epicardial EVs received one intracardiac injection into the observable infarct border zone (EV titre of 10^9^, in a volume of 5 uL, amount of EV within the range previously used in the literature^28^) or phosphate-buffered saline (PBS) (vehicle) at the time of the injury. In order to maximize the amount of particles injected, several tests were performed injecting larger volumes or administering more than one injection around the injured area in neonatal hearts. Unfortunately, both of these tests reduced the survival rate of pups after surgery. Therefore, a compromise was reached to inject the largest amount of EVs that enabled a good survival rate, and still fall within the reported dosing in the literature.^28^ Whole hearts were then excised at 48 h, 7 and 21 days following MI, which correspond to Days 2, 7, and 21 post-injury. Hearts collected for immunostaining were washed in ice-cold PBS prior to overnight fixation in a 2% formaldehyde solution at 4°C.

### 2.3 Immunodetection methods

Cultured cells from neonatal mice or hES-CM were fixed at 4°C overnight in 2%PFA prior to staining. Cryosections were processed for indirect immunofluorescence using standard methods. Primary antibodies are listed in Supplementary *Table S2*. Alexa Fluor secondary antibodies (Invitrogen, 1:200) were used in all cases. Imaging was performed using an Olympus FV1000 confocal microscope (tissue sections/cell culture) and Leica SP8 Navigator. Images were digitally captured and processed using Fiji software.

### 2.4 Histology

On Day 21 after MI, hearts were harvested and processed for paraffin embedding, cut into Superfrost slides, and deparaffinized using standard methods. For Masson’s trichrome staining (Abcam), sections were stained according to the manufacturer’s instructions. For Picrosirius red staining, sections were stained using the Picro Sirius Red Stain Kit (Abcam) for 60 min according to the manufacturer’s protocol and digitalized using a scanner. Picrosirius staining was also imaged under polarized light using a Nikon 3i 4× and 20× objectives. For FUCCI sections, tissue was co-immunostained with the cardiomyocyte marker α-actinin. For subsequent analyses, only cardiomyocytes expressing α-actinin were taken-into-account. Total of 10–15 sections per heart were quantified, at an average of 900 cells throughout each section.

### 2.5 Cell culture

H9C2 cells (ATCC) and mouse epicardial cells^36^ according to standard procedure.

Epicardial cells were cultured maintaining epithelial morphology until 80% confluency in T25 (2.5 10^6^) or T75 (8 10^6^) flasks according to procedures described upon line generation.^36^ Prior to EV isolation, cells were cultured in OPTIMEM without serum for 48 h. Treatment with epicardial EVs (both for H9C2 cell line and neonatal primary cardiomyocytes) was performed for 24 h (at a concentration of 1.5 × 10^8^ EVs, consistent with previously described doses^28^^,^^37^). Where applicable, bromo deoxyuridine (BrdU) administration was also added at 50 ng/mL for 24 h. H9C2 cells were seeded (at an initial density of 0.4x10^6^) in P12 plates on coverslips prior to treatment.

### 2.6 EV isolation and analysis

EVs were isolated from conditioned media (OPTIMEM without serum) by ultracentrifugation following standard procedures.^38^ Briefly, cells were cultured in OPTIMEM for 48 h prior to collection of CM. EV isolation was performed following an initial centrifugation step of the conditioned media at 2000*g* to remove cellular debris followed by filtration through a 0.22 um filter. Resulting supernatant was subjected to ultracentrifugation of 1 h at 120 000*g* followed by a second ultracentrifugation of 1 h at 120 000*g* after washing the pellet with PBS. Analyses of vesicle size were performed by Nanoparticle-tracking assays (Nanosight). Labelling of EVs was performed with PKH26 (Sigma) according to the manufacturer’s directions.

### 2.7 RNA isolation and gene expression profile by qRT–PCR

Total RNA from cultured cells and epicardial EVs prior to sequencing was isolated using Trizol reagent (Invitrogen) according to the manufacturer’s instructions. Total RNA was reverse transcribed using oligo-dT primers and Superscript III RT (Invitrogen). qRT–PCR analysis was performed on a ViiA 7 Real-Time PCR System (Thermo Fisher Scientific) using Fast SYBR Green Master Mix (Thermo Fisher Scientific). Data were normalized to *Gapdh* housekeeping gene expression. Fold changes in gene expression were determined by the 2^−ΔΔCT^ method. Statistical differences were detected using an unpaired, 2-tailed Student’s *t-*test.

### 2.8 Tem processing and immunogold labelling

EVs were resuspended in TBS-3% PFA and adsorbed to the grids for 5 min. Primary antibody CD63 (Santa Cruz) was used at a 1/50 dilution and then the grid was placed for 60 min on a drop of secondary antibody coupled with 10 nm diameter gold particles diluted 1/40. The grids were negatively stained with 2% uranyl acetate for 10 s.

### 2.9 Neonatal cardiomyocyte isolation and culture

P1 pups were sacrificed by cervical dislocation followed by decapitation and hearts dissected for cardiomyocyte isolation using Pierce Primary cardiomyocyte isolation kit according to manufacturer’s directions. Digested cardiomyocytes were seeded (at a density of 0.25 × 10^6^) in P12 well plates on coverslips. After 4 days in culture, neonatal cardiomyocytes were treated with epicardial EVs. Treatment with EVs was performed for 24 h (1.5 × 10^8^ particles). Where applicable, BrdU was added for 24 h at 50 ng/mL.

### 2.10 Epicardial isolation from human right atrial appendage

Right atrial appendage (RAA) from patients undergoing bypass surgeries were dissected for epicardial and EPDC culture according to References.^39^ All patients provided written informed consent and the investigation conformed to the principles outlined in the Declaration of Helsinki.

### 2.11 H9 human embryonic stem cell-derived epicardial differentiation

Human H9 stem cells (H9, WAe009-A; and WiCell) were differentiated to epicardium following a published protocol for epicardial differentiation.^40^

### 2.12 Generation and cryoinjury of EHM^41^

EHM were generated as previously described.^33^ Briefly, hES2-LUC^+^ derived cardiomyocytes and human foreskin fibroblasts (70:30 ratio) were interspersed within a bovine collagen hydrogel and cast into circular silicone moulds. These circular EHM were transferred to flexible stretchers 3 days post-casting that permitted auxotonic spontaneous contractions. EHM were maintained in culture for 28 days and cryoinjuries performed thereafter. Cryoinjuries were performed utilizing a liquid nitrogen-cooled stainless-steel oral gavage attached to a plastic syringe barrel. Injuries to only one separated arm of each EHM were performed twice to ensure a under control conditions irreversible injury.

### 2.13 Assessment of EHM contractile function

At specific time points post-cryoinjury (Cry), EHM were mounted onto isometric force transducers (Föhr Medical Instruments, Germany), lowered into 37°C water-jacketed organ baths and bathed in Tyrode’s solution (in mM: 119.8 NaCl, 5.4 KCl, 1.05 MgCl_2_, 0.42 NaH_2_PO_4_, 22.6 NaHCO_3_, 5 glucose, 0.28 L ascorbic acid). The EHM were gradually stretched according to the Frank–Starling mechanism in the presence of 1.8 mM Ca^2+^ to determine the maximal force of contraction (*F*_max_). All subsequent functional assessments were performed at *F*_max_.

### 2.14 Epicardial EV sequence and bioinformatics

EV RNA isolation was obtained via AllPrep DNA/RNA Mini Kit*.* The AllPrep DNA/RNA Mini kit (Qiagen, catalogue number: 80 204) was used to extract exosomal RNAs following the manufacturer’s protocol. RNA was quantified using the Nanodrop. Quality and integrity of the start material was assessed with the Fragment Analyzer from Advanced Analytical by using the standard sensitivity RNA Analysis Kit (DNF-471).

Library preparation was performed using TrueSeq Kit (*TruSeq Small RNA Library Prep Kit Illumina Cat N°* RS-200-0012) with modifications on gel size selection (30–300 bp fragments). For accurate quantitation of exosome libraries, a fluorometric based system, the QuantiFluor™dsDNA System from Promega were used. The size of final libraries was determined by using the dsDNA 905 Reagent Kit (Fragment Analyzer from Advanced Bioanalytical) exhibiting a sizing of 30–350 bp in average. Libraries were pooled and sequenced on the Illumina HiSeq 4000 (SE; 1 × 50 bp; 10–15 Mio reads/sample).

Bioinformatic analysis was performed using Oasis.^42^ Sequence reads were pre-processed to remove adapters and retain only 16–30 bp length reads. Reads were aligned to reference genome. The reads aligning to each known mature miRNA were counted using Bioconductor packages for next-generation sequencing data analysis based on miRNA definitions in miRBase database. The differential expression analysis between different sample types was performed using the negative binomial statistical model of read counts as implemented in the DESeqBioconductor package. The statistical significance of differential expression is established based on the false discovery rate (FDR)-adjusted *P*-values. The cluster analysis of all differentially expressed miRNA was performed using the Bayesian infinite mixture model. Pathway enrichment analysis was conducted with Gene Ontology. Enrichment *P*-values were corrected for FDR and were considered significant when adjusted *P*≤0.05.

GEO accession number: GSE161630 (https://www.ncbi.nlm.nih.gov/geo/query/acc.cgi?acc=GSE161630).

### 2.15 AAV vectors

All of the AAV vectors used in this study were generated by the International Centre for Genetic Engineering and Biotechnology AAV Vector Unit (http://www.icgeb.org/avu-core-facility.html) using a dual-triple plasmid co-transfection procedure followed by polyethylene glycol precipitation and purification through CsCl2 gradient centrifugations as described previously. AAV particles were used to infect the cells at a MOI of 10^4^ for 48 h before fixing for further analysis.

### 2.16 AAV treatment of EHM

EHMs were infected immediately after cryoinjury (as in Section 2.12) with AAV serotype 9 (AAV9)-miR100 or AAV9-miR30a with a MOI of 10^4^. Fresh medium was replenished 24 h later and then every 48 h until contractile assessment on Day 7 (D7) Cry (as in Section 2.13).

### 2.17 Western blotting

Primary neonatal cardiomyocytes were homogenized using RIPA buffer in presence of protease and phosphatase inhibitors [Inhibitor cocktail (11 697 498 001, Roche, Switzerland) and 1 μM sodium orthovanadate]. After clarification by centrifugation, protein concentration was measured using Pierce BA Protein Assay kit (23 227, Thermo Scientific; USA) following manufacturer instructions. Protein (30 μg) were denaturized at 95°C for 5 min, loaded on an 8% polyacrylamide SDS-PAGE gel and run at 120 V for 90 min. Subsequently, samples were transferred to a nitrocellulose membrane by wet transfer at 400 mA for 2 h. Membranes were blocked with 5% BSA for 1 h and incubated with primary antibodies O/N at 4°C. Next day, membranes were washed in TBS-T buffer and incubated with the corresponding HRP-conjugated secondary antibodies (Dako, Denmark) at 1:5000 dilution for 1 h at RT. After washing, signal was developed using ECL Primer Western Blotting Detection Reagent (Amersham; UK) and detected by a LAS-3000 imaging system (Fujifilm; USA). The primary antibodies used were diluted 1:1000.

### 2.18 Statistics

Statistical analyses were performed using GraphPad Prism 7 software. The statistical significance between two groups was determined using a non-parametric Mann–Whitney test or an unpaired two-tailed Student’s *t-*test; these included an *F* test to confirm the two groups had equal variances, and the data were reported as mean ± SEM. Among three or more groups 1-way ANOVA followed by Tukey’s multiple comparisons test was used. *P* ≤ 0.05 was considered statistically significant.

## 3. Results

### 3.1 Mouse epicardial cells release EVs, which are taken up by cardiomyocytes and promote cell cycle activity

**Figure 1:**
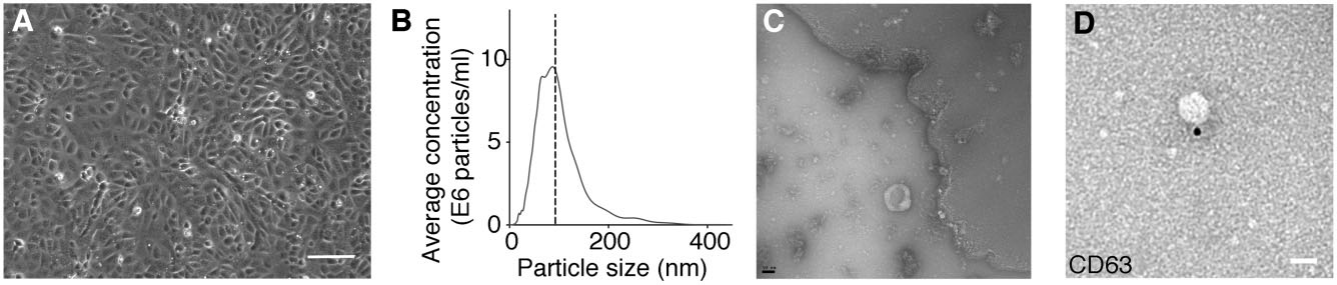
EVs isolated from epicardial conditioned media. (*A*) Brightfield image of mouse epicardial cells in culture with characteristic epithelial morphology. Scale bar denotes 100 µm. (*B*) Representative size characterization of EVs by nanoparticle tracking. This example shows a particle mode of 93 nm size. (*C*) Transmission electron micrograph of EVs isolated from epicardial conditioned media. Note the cupped-shaped morphology and size (80 nm). Scale bar denotes 50 nm. (*D*) CD63 immunogold labelling of EVs isolated from epicardial conditioned media. Data in (*B*) is presented as frequency of EVs in a given size. Scale bar denotes 50 nm.

There is extensive evidence that epicardial cells play a major role in cardiomyocyte proliferation during development and regeneration through paracrine signalling (reviewed in Reference^10^). This led us to determine whether the epicardium communicates with cardiomyocytes *via* EV shedding. To begin to address this, we cultured immortalized mouse embryonic epicardial cells from a previously established cell line^36^ (*Figure 1A*) and isolated EVs from conditioned medium utilizing a previously described ultracentrifugation protocol.^38^

We identified EVs in the epicardium conditioned medium that displayed typical exosomal characteristics, such as a modal particle size of 98 nm (analysed by Nanosight and TEM) (*Figure 1B and C* and Supplementary *Figure S1A–C*), canonical morphology and expression of the exosomal marker CD63 (*Figure 1D*).

To assess the potential function of the epicardial EVs *in vitro*, we first screened for proliferation-inducing effects in the well characterized H9C2 rat myocyte cell model.^43^ Exposure of H9c2 cells to isolated mouse epicardial EVs (mepic EV) resulted in significantly increased level of cell cycle activity, by more than two-fold as reported by phosphohistone H3 staining (PH3) PBS 1.81±0.17; mepic EV 5.13±2.10, *P*≤0.01) (Supplementary *Figure S2A–C*). To determine EV uptake, we labelled EVs with the membrane dye PKH26 and confirmed intracellular fluorescent expression in target H9C2 cells (Supplementary *Figure S2D*). Interestingly, PHK26^+^ EVs were observed localizing predominantly in PH3^+^ cycling cells (Supplementary *Figure S2E and E’*).

### 3.2 Epicardial EVs promote cell cycle activity in mouse cardiomyocytes *ex vivo*

**Figure 2:**
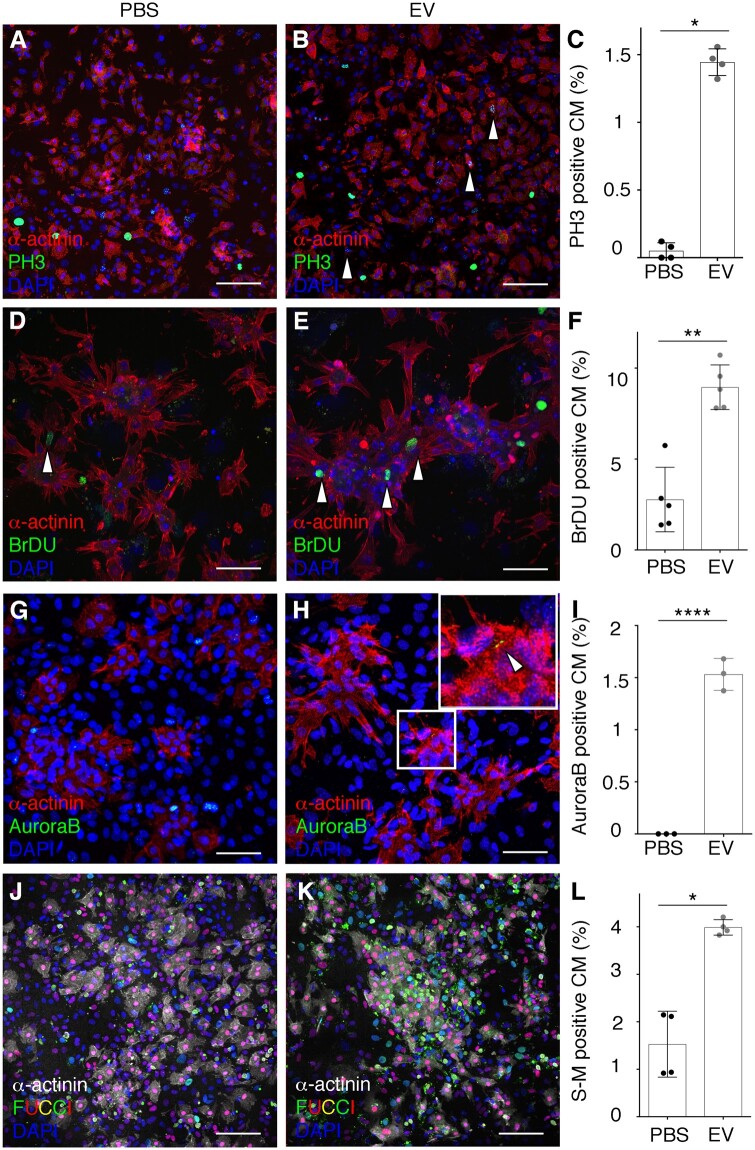
Epicardial EVs promote cell proliferation in mouse neonatal cardiomyocytes. (*A, B*) Immunostaining of PH3 (green) and α-actinin (red) of cultured mouse neonatal cardiomyocytes treated with vehicle (PBS) (*A*) or epicardial EVs (EV) (*B*). Arrowheads in (*B*) point to PH3 positive cardiomyocytes, co-stained for both markers. (*C*) Quantification of proportion of cardiomyocytes (α-actinin positive) positive for PH3 in both vehicle (PBS) and EV-treated cultures (EV). (*D, E*) Immunostaining for BrdU (green) and α-actinin (red) of cultured mouse neonatal cardiomyocytes, treated with vehicle (PBS) (*D*) or epicardial EVs (EV) (*E*). (*F*) Quantification of proportion of cardiomyocytes (α-actinin positive) positive for BrDU in both vehicle (PBS) and EV-treated cultures (EV). (*G, H*) Immunostaining for Aurora-B (green) to show cytokinesis and α-actinin (red) of cultured mouse neonatal cardiomyocytes, treated with vehicle (PBS) (*G*) or epicardial exosomes (Exo). (*H*) Magnification of boxed area and arrowhead in (*H*) point to a positive cytokinetic furrow between cardiomyocyte cells. Note only immunoreactivity during cytokinesis is considered as positive Aurora-B signal. (*I*) Quantification of proportion of cardiomyocytes (α-actinin positive) positive for PH3 in both vehicle (PBS) and EV-treated (EV) cultures. (*J, K*) Immunostaining α-actinin (grey) of cultured mouse neonatal cardiomyocytes from FUCCI line, in which endogenous Venus (green) fluorescence labels cells in S-M and Tomato, (red), cells in G1 phase of the cell cycle; treated with vehicle (PBS) (*J*) or epicardial EVs (EV) (*K*). (*L*) Quantification of proportion of cardiomyocytes (α-actinin positive) positive for Venus-FUCCI in both vehicle (PBS) and EV-treated (EV) cultures. DAPI (blue) labels cell nuclei. Data are presented as mean±SEM. *n*=4 PH3, Control; *n*=4 PH3, EVs; *n*=5 BrdU, Control; *n*=5 BrdU, EVs; *n*=3 Aurora-B, Control; *n*=5 Aurora-B, EVs; *n*=4 FUCCI, Control; *n*=4 FUCCI, EVs. *P<0.05, **P<0.01, ***P<0.001, ****P<0.0001. Mann–Whitney test. Scale bars: 50 µm.

To further interrogate the effect on cardiomyocyte cell cycle activity, we treated primary mouse cardiomyocytes isolated at P1 with mouse epicardial-derived EVs. We detected PH3^+^ cardiomyocytes (co-stained with α-actinin) (*Figure 2A–C*) and the successful incorporation of BrdU (*Figure 2D–F*) consistent with previous reports of active proliferation at this stage^13^^,^^32^ and revealed both cell cycle markers exhibited a significant three-fold increase in expression relative to controls (PH3: PBS 0.05±0.05; EV 1.45±0.09, *P*≤0.05; BrdU: PBS 2.77±1.78; EV 8.9±1.23, *P*≤0.01). We also detected an increase in Aurora-B kinase, indicating that elevated cell cycle activity was partially concomitant with increased cell division as revealed by a mitotic cleavage furrow (PBS 0±0; EV 1.53±0.15, *P*≤0.0001) (*Figure 2G–I*).

We further analysed cell cycle progression and activity by taking advantage of the FUCCI reporter mouse line.^34^^,^^44^ In the FUCCI reporter, cells progressing through the cell cycle are labelled with fluorescent Cherry when in G1 (Cherry reports ubiquitination domains of Cdt1) and with the Venus fluorescence when transitioning through S phase and mitosis (Venus reports ubiquitination domains of Geminin).^34^ Treatment of primary neonatal cardiomyocytes isolated from FUCCI hearts revealed an increase in cardiomyocytes that were undergoing S phase and mitosis when treated with mepic exo (PBS 1.53±0.69; EV 3.98±0.16, *P*≤0.05) (*Figure 2J–L*). We then sought to analyse downstream pathways that were mediating the increase in cell cycle activity in murine neonatal cardiomyocytes. We investigated activation of ERK, AKT, and Hippo pathways, all of which have been previously implicated in cell cycle activity and proliferation in cardiomyocytes.^45–48^ Following treatment of cardiomyocytes with epicardial EVs, we detected a significant increase in phosphorylation of Akt and a trend in the reduction in (inactive) phosphorylated Yap, favouring the non-phosphorylated (activated) Yap isoform, albeit this was not significant. We did not detect a significant increase in the phosphorylation of ERK, but observed a trend towards activation of the ERK pathway followed epicardial EV treatment (Akt: PBS: 0.63±0.03, EV: 2.071±1.3, *P*<0.05; Yap PBS: 1.26±1.2, EV: 7.8±8.1, *P*=0.0734; Erk PBS: 0.91±0.857, EV: 2,38±2.372, *P*=0.34) (Supplementary *Figure S3*). Taken together, these data revealed that epicardial-derived EVs significantly induced a pro-proliferative response in murine neonatal cardiomyocytes *ex vivo* and that this response is mediated by activation of the Akt pathway and partial activation of Hippo and ERK signalling pathways.

### 3.3 Epicardial EVs induce a proliferative response *in vivo* during and beyond the murine neonatal regenerative window

The regenerative capacity of the mouse neonatal heart has been described in injury models of apical resection,^13^ cryoinjury,^49^^,^^50^ and left anterior descending^35^ artery ligation.^51^ The extent of regeneration is linked with the proliferation of pre-existing cardiomyocytes^13^ and angiogenesis,^52^ similar to that described in lower vertebrates.^11^ However, this regenerative potential is lost within the first week of life by P7 correlating with cardiomyocytes exiting the cell cycle.^13^ Injured P7 hearts, therefore, behave comparably to adult mammalian hearts, regarding their response to injury; after MI a scar is formed impeding regeneration and cardiomyocytes are unable to proliferate and replace the loss myocardium.^13^^,^^31^^,^^32^

**Figure 3:**
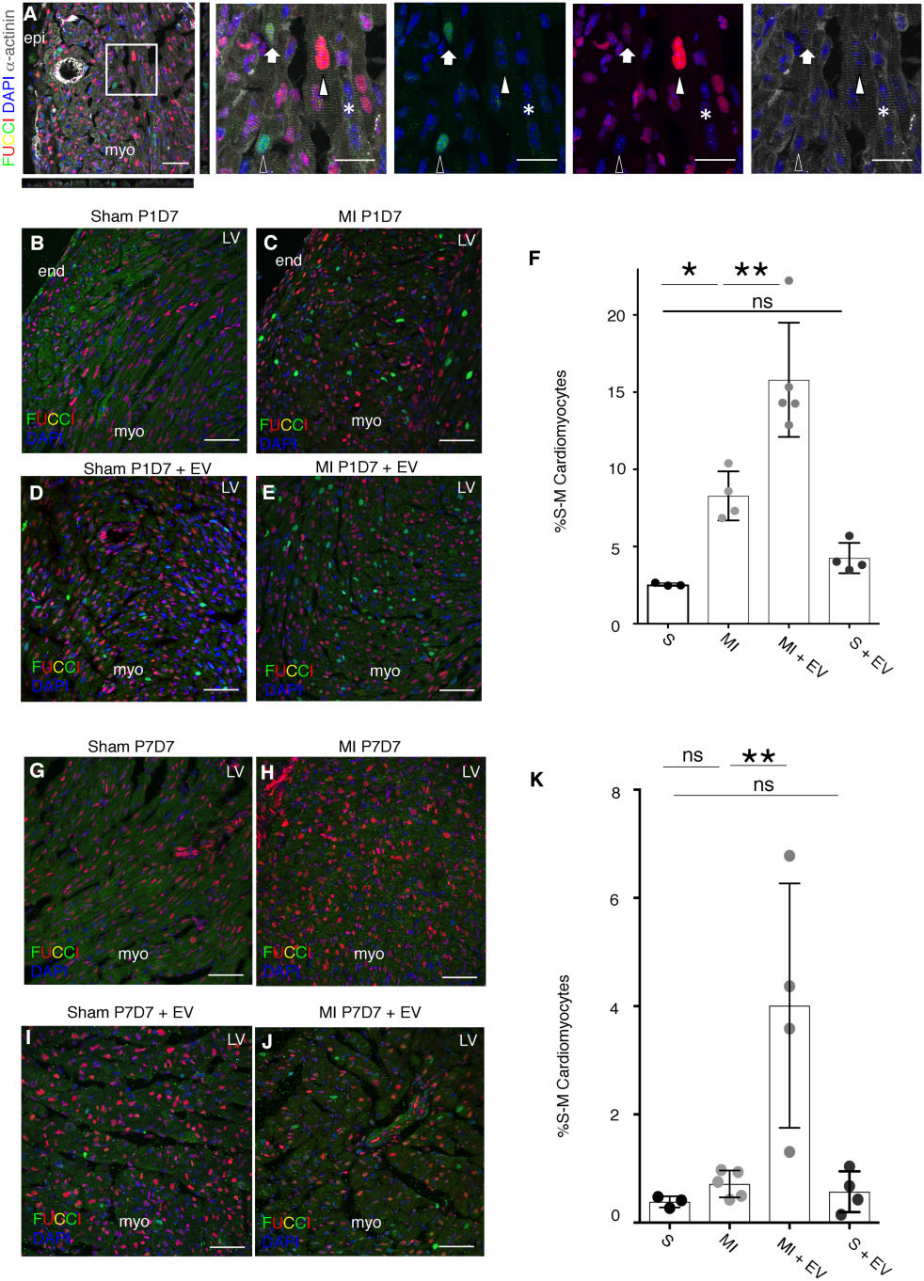
Epicardial EVs increase cardiomyocyte proliferation after injury in P1 and P7 hearts. (*A*) α-actinin (grey) and FUCCI reporter (green and red) detection in the neonatal heart. DAPI (blue) labels cell nuclei. Left panel shows merge of all the channels and includes orthogonal views to highlight detection of cardiomyocyte nuclei positive for the FUCCI reporters. Middle and right panels are separate channels from the magnified boxed area in the left panel. Empty arrowhead points to a Venus (green) positive cardiomyocyte. Filled arrowhead points to a tomato (red) positive cardiomyocyte. Filled arrow points to a cardiomyocyte displaying both FUCCI markers. Asterisk points to a cardiomyocyte that is not labelled for any of the FUCCI reporters. (*B*–*E*) FUCCI cell cycle reporter (red and green) and DAPI to label cell nuclei are shown in P8 heart sections collected 7 days after sham or MI procedure followed by epicardial EV or vehicle injection performed at P1. (*B*) Sham, (*C*) MI, (*D*) Sham with administration of EVs, (*E*) MI with administration of EVs. (*F*) Quantification of cardiomyocytes’ cell cycle reported by FUCCI expression at P8, 7 days after procedure at P1 (S: sham, MI: myocardial infarction, MI+ EV: myocardial infarction followed by EV injection, S+EV: Sham + EV injection). (*G*–*J*) FUCCI cell cycle reporter (red and green) and DAPI to label cell nuclei are shown in P14 heart sections collected 7 days after sham or MI procedure followed by exosome or vehicle injection performed at P7. (*G*) sham, (*H*) MI, (*I*) sham with administration of exosomes, (*J*) MI with administration of exosomes. (*K*) Quantification of cardiomyocytes’ cell cycle reported by FUCCI expression at P14, 7 days after procedure at P7 (S, sham; MI, myocardial infarction; MI+ EV, myocardial infarction followed by EV injection; S+EV, Sham + EV injection; LV, left ventricle; LA, left atria; RV, right ventricle; RA, right atria; epi, epicardium; myo, myocardium; end, endocardium). Data are presented as mean SEM. *n*=3 ShamP1D7; *n*=4 MI P1D7; *n*=5 MI+EVsP1D7; *n*=4, Sham+EVsP1D7; *n*=3 ShamP7D7; *n*=5 MI P7D7; *n*=4 MI+EVsP7D7; *n*=4, Sham+EVsP7D7. *P<0.05, **P<0.01, ***P<0.001. Mann–Whitney test. Scale bars: 50 µm except magnified panels in (*A*): 20 µm.

To test the regenerative capacity of epicardial EVs in murine hearts, we induced a MI by LAD ligation in regenerative P1 and non-regenerative P7 FUCCI R26Rp mouse pups (in which FUCCI expression is directed by the Rosa26 promoter)^34^ followed by intracardiac injection of epicardial EVs into the muscle wall of the left ventricle, around the injury area. Expression of the FUCCI reporter in cardiomyocytes was assessed by co-detection of α-actinin throughout orthogonal views (*z*-axis) per section. Only nuclei clearly contained within an α-actinin+ cardiomyocyte were considered for quantification (*Figure 3A*).

Analysis of FUCCI cell cycle progression in P1 mouse hearts 1 week post-MI showed an increase in proliferation, relative to sham-operated control hearts without any treatment (*Figure 3B, C and F* and Supplementary *Figure S4A and C*). Intracardiac injection of EVs in sham controls (S) at the time of the surgery failed to invoke a significant proliferative response (*Figure 3D and F* and Supplementary *Figure S4B*). However, injection of EVs after MI [MI plus EV (MI+EV)] elicited an organ-wide response in terms of increased cardiomyocyte cell cycle activity and a doubling of the number of proliferative cardiomyocytes, compared to injured hearts injected with vehicle-alone (MI) (S 2.58±1.01; MI 8.28±1.58, *P*<0.05; MI+EV 18.8±3.69, *P*<0.01; S+EV 4.25±0.98) (*Figure 3E and F* and Supplementary *Figure S4D*). Remarkably, treatment of non-regenerative P7 mouse hearts 7 days post-MI also revealed a significant increase in proliferating cardiomyocytes when comparing MI alone with MI+EV treatment (*Figure 3G, H and K* and Supplementary *Figure S5A and C*) and sham hearts injected with EVs (S+EV) (S 0.38±0.1; MI 0.72±0.10; MI+EV 4.01±2.25, *P*<0.01; S+EV 0.57±0.37) (*Figure 3I and K* and Supplementary *Figure S5B*). Cumulatively, these results suggest that the innate cardiomyocyte proliferative response in P1 mouse hearts after acute injury can be further enhanced by exposure to epicardial-derived EVs and significantly, that the relatively quiescent, more adult-like cardiomyocytes at P7, can be induced to re-enter the cell cycle by EV treatment.

### 3.4 Epicardial EVs fail to promote a longer-term regenerative response in P7 neonatal hearts post-MI

In order to assess the longer-term effects of epicardial EV injection on non-regenerative P7 mouse hearts, we analysed scar formation 21 days after P7 injury and EV treatment. Histological examination showed no significant differences in scar size in the left ventricle upon MI in those hearts injected with vehicle or epicardial EVs (PBS injected, 11.99±3.23; EV injected, 15.02±3.32) (Supplementary *Figure S6A–C*). To further explore the nature of the scarring response, we analysed Picrosirius Red staining under polarized light and detected no significant differences in the amount or basic composition of the scar formed after injury with injection of mouse epicardial-derived EVs (PBS injected 9.16±4.22; EV injected 7.51±6.13) (Supplementary *Figure S6D and E*).

We then sought to further characterize the longer-term effects of EVs and analysed whether they elicited a pro-angiogenic response in P7 infarcted hearts. We did not observe significant differences in the vascular density after injury or EV treatment (PBS injected 9.25±1.14; EV injected 8.76±0.77) (Supplementary *Figure S6F and G*). Moreover, analysis of cardiomyocyte sarcomeric structure by α-actinin staining showed no obvious changes in extent of myocardial disarray. Taken together, these results show that whilst we observed early effects (within the first 7 days) on cardiomyocyte proliferation (*Figure 3*) (Supplementary *Figures S4 and S5*), there was no long-term beneficial effect of epicardial EV injection in response to MI in the non-regenerative, adult-like P7 setting; suggesting that increased cardiomyocyte proliferation alone is insufficient to promote optimal repair, longer-term regeneration or improved outcome.

### 3.5 Human primary and embryonic stem cell-derived epicardial cells secrete EVs that enhance proliferation in mouse and human cardiomyocytes

**Figure 4:**
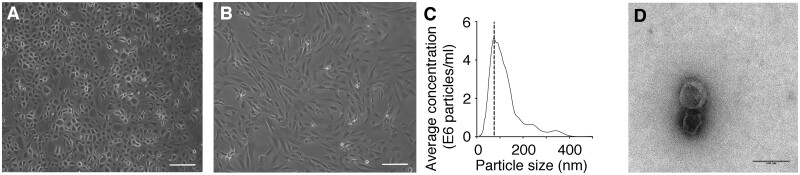
Epithelial and mesenchymal human epicardial cells signal through EV release. (*A*) Brightfield image of human epicardial cells in culture after expansion from patient RAA biopsy, displaying epithelial morphology. (*B*) Brightfield image of human epicardial cells in culture after expansion from patient RAA biopsy, displaying mesenchymal spindle morphology after spontaneous activation *in vitro*. (*C*) Size characterization of EVs by nanoparticle-tracking graph showing a mode of particle size of 93 nm. (*D*) TEM image of EVs isolated from epicardial conditioned media. Note the cupped-shaped morphology and size (80 nm). Data in (*C*) is presented as frequency of EVs in a given size. Scale bars: (*A*) 100 µm; (*D*) 100 nm.

To determine whether our findings in mice might be conserved in humans, we sought to determine whether EVs could be isolated from epicardial cells from RAA biopsies from patients undergoing coronary artery bypass graft surgery^39^ and differentiated H9 human embryonic stem cells.^40^ EPDCs from RAA biopsies were expanded with a cobble-stone (inactive) epithelial morphology (*Figure 4A*) and underwent spontaneous, or evoked, activation to a more spindle-like, mesenchymal form that recapitulates epithelial-mesenchymal transition^39^ (EMT) (*Figure 4B*). EMT has also been observed to inherently occur with epicardial activation in injured adult mouse hearts.^53^ Human primary EPDCs secreted exosomal-like EVs that were within the expected size range (*Figure 4C*) and displayed a characteristic cupped-shaped morphology (*Figure 4D*). Similarly, hESC-derived epicardial cells also displayed a cobble-stone-like morphology (Supplementary *Figure S7A*) and strongly expressed epicardial markers Wt1, Tbx18, and Tcf21 (Supplementary *Figure S7A–D*^40^). Analysis of their cell-conditioned media revealed particles that were within the size range and morphological appearance for EVs (Supplementary *Figure S7E*).

### 3.6 Human primary EPDC-derived EVs induce proliferation of cardiomyocytes in mouse and human 2D cardiomyocyte cultures and in EHM after cryoinjury with improved functional outcome

To explore the functional potential of human epicardial EVs, we initially tested the effect of the more activated, mesenchymal (spindle) EPDC-derived EVs on primary mouse cardiomyocytes expressing the FUCCI cell cycle reporter. Spindle EPDC-derived EVs significantly increased the number of cardiomyocytes undergoing cell cycle activity (PBS 1.34±0.73; hEPDC EV 6.44±1.87, *P*≤0.01) (Supplementary *Figure S8A–C*). Concurrently, incubation of spindle EPDC-derived EVs with human ES-derived cardiomyocytes led to a significant increase in PH3^+^ cells and BrdU incorporation (PH3: PBS 0.317±0.04; hEPDC EV 1.75±0.13, *P*≤0.0001; BrdU: PBS 3.93±0.33; hEPDC EV 8.46±0.43, *P*≤0.05) (Supplementary *Figure S8D–I*).

**Figure 5:**
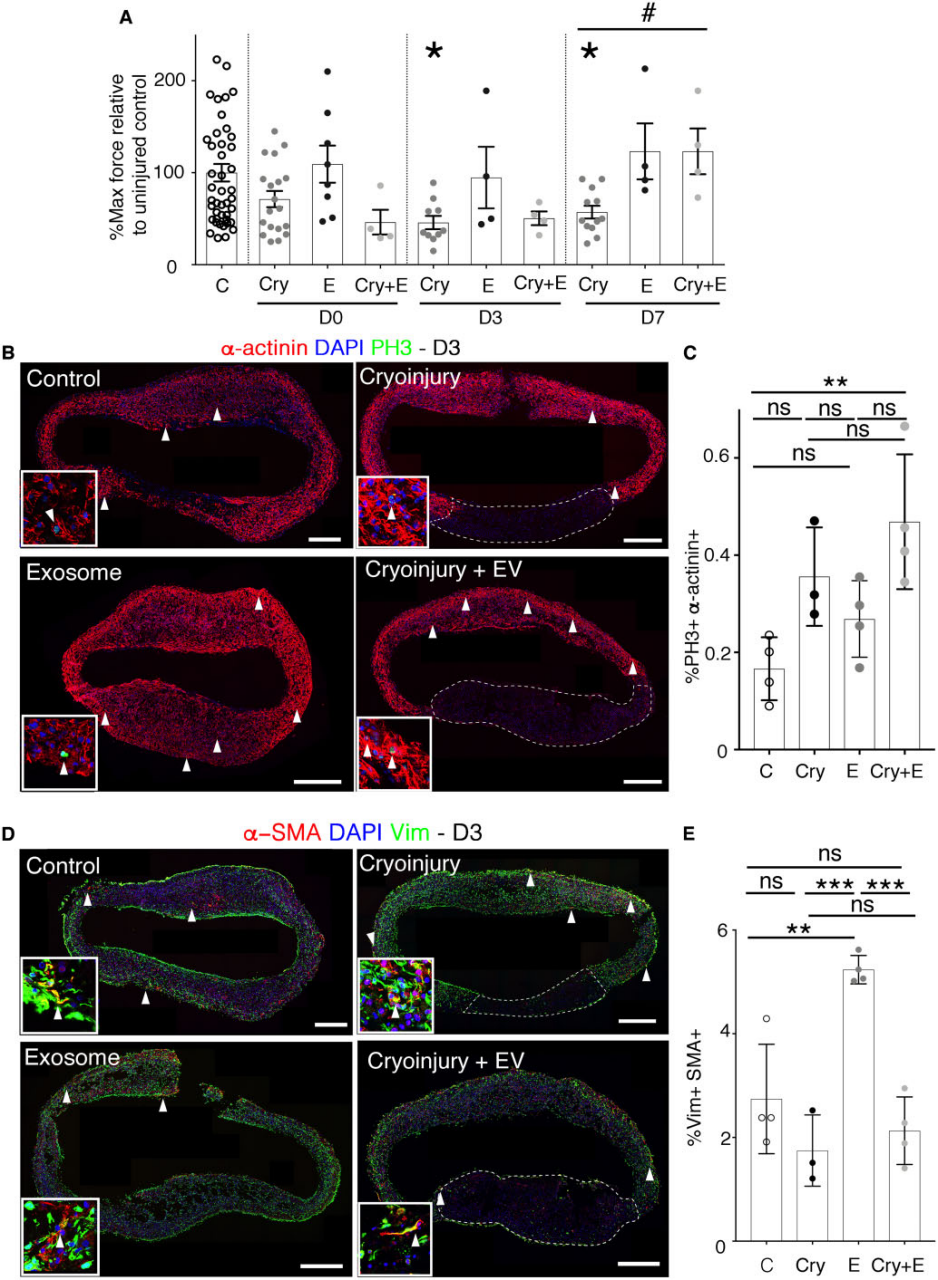
Enhanced functional regeneration of EHM after cryoinjury with human epicardial EV treatment. (*A*) Quantification of maximum contraction forces of EHM after cryoinjury and treatment with human epicardial EVs at day of procedure (d0), 3 days post-procedure (d3), and 7 days post-procedure (d7). (*B*) α-actinin (red) and PH3 (green) confocal images of EHMs at Day 3 after procedure for the four experimental groups. Arrowheads point to PH3 positive cardiomyocytes. Boxed areas show magnification of representative cells displaying co-staining of PH3 and actinin. Dashed lines surround the cryoinjured EHM arm, displaying cardiomyocyte loss. (*C*) Graph showing the percentage of cardiomyocytes expressing PH3 in EHMs (C, control; Cry, cryoinjured; E, EVs; Cry+E, cryoinjured treated with EVs). (*D*) α -SMA (red) and vimentin (green) staining of EHMs 3 days after procedure. Arrowheads point to a-SMA positive fibroblasts (vimentin positive cells). Boxed areas show magnification of representative cells displaying co-staining of vimentin and a-SMA. (*E*) Quantification of the percentage of vimentin cells co-expressing α -SMA in EHMs (C, control; Cry, cryoinjured, E, EVs; Cry+E, cryoinjured treated with EVs). Data are presented as mean±SEM. *n*= 4 control d3; *n*=3 EV-treated d3; *n*=4 cryoinjured d3; *n*=4 cryoinjured EV-treated d3; *n*=4 control d3; *n*=3 EV-treated d3; *n*=4 cryoinjured d3; *n*=4 cryoinjured EV-treated d3. For the maximum force analysis *n* =44 control *n*=8 EV d0 *n*=19 cryo d0 *n*=4 cryo+EV d0; *n*=4 EV d3 *n*=10 cryo d3 *n*=4 EV+cryo d3; *n*=4 EV d7 *n*=12 cryo d7 *n*=4 EV cryo d7. *P<0.05, for post ANOVA comparisons with control group #*P*<0.05 for *t*-test for differences between cryoinjured (Cry) and cryoinjured treated with human epicardial EVs (Cry+E). Scale bars: 200 µm. Dashed lines in (*B*) and (*D*) right panels show the loss of cardiomyocytes upon cryoinjury.

Next, we employed the EHM model^41^ with properties of postnatal human hearts^33^ to assess structural and functional consequences of epicardial EV exposure. To simulate myocardial damage, we established a cryoinjury EHM model (Supplementary Video S1 and *Figure S9A and B*) and treated with human EPDC-derived EVs to determine effects on functional outcome (force generation). Cryoinjury itself induced ≈40%–60% decrease in force generation, that was sustained until D7 post-injury [Day 3 (D3) Cry 46%±23% of uninjured control (C), D7 Cry 57%±24%, *P*≤0.05] (*Figure 5A*) concomitant with the loss of cardiomyocytes (*Figure 5B*, right panels). This impaired function was detected in all cryoinjured groups irrespective of EV treatment until D3 post-injury (*Figure 5A*). At D7 Cry, functional restoration to levels comparable in uninjured controls was detected in cryoinjured EHMs acutely treated with human EPDC-derived EVs. EV (+E) treatment in uninjured EHM did not significantly alter functionality (D7 Cry 57%±24%; D7 Cry+E 123%±50%, *P*≤0.05) (*Figure 5A*).

The acute treatment of cryoinjured EHM with EVs induced a significant increase in cardiomyocyte proliferation three days post-injury, when compared with baseline (intact EHM) controls, albeit not significantly different to that induced by cryoinjury alone [Control (C) 0.16±0.06; Cry 0.35±0.10; EV (E) 0.27±0.08; Cry+E 0.47±0.14, *P*≤0.01) (*Figure 5B and C*). At 7 days Cry, the initial cardiomyocyte proliferative response in EHMs declined and no further response could be detected, irrespective of injury or epicardial EV treatment (C 0.12±0.10; Cry 0.10±0.07; E 0.11±0.09; Cry+E 0.15±0.13) (Supplementary *Figure S9C and D*).

Interestingly, EVs alone evoked a strong increase in α-smooth muscle actin (α-SMA) expression in the fibroblast population at D3 after cryoinjury relative to vehicle -treated controls, indicating an activation of myofibroblasts^54^ in the absence of an external inflammatory stimulus (C 2.74±1.05; Cry 1.74±0.69; E 5.25±0.08; Cry+E 2.13±0.65 *P*≤0.01 for EV treatment vs. control, *P*≤0.001 for EV treatment vs. cryoinjury and Cryoinjury plus EVs vs. EVs alone) (*Figure 5D and E*). Following injury there was a delayed increase in myofibroblast α-SMA expression, however, this was not dependent upon EV treatment (C 3.07±0.68; Cry 7.82±2.63; E 7.82±2.63; Cry+E 6.25±1.23, *P*≤0.05, for cryoinjury plus EVs vs. EVs alone and *P*≤0.01 for cryoinjury vs. control and EV treatment vs. cryoinjury) (Supplementary *Figure S9E and F*).

These data collectively reveal that human epicardial EVs can stimulate an initial increase in cardiomyocyte proliferation in a model of injured human myocardium, which manifests longer term as improved contractile function.

### 3.7 Mouse and human epicardial EVs carry a conserved miRNA cargo

**Figure 6:**
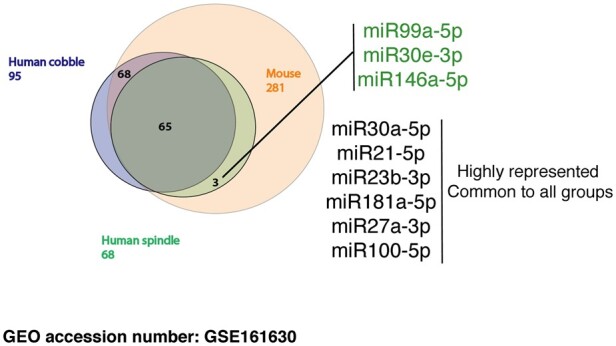
miRNA cargo in epicardial EVs is highly conserved in mouse and human; but slight differences are observed among spindle and cobble human epicardial exosomes. (*A*) Venn diagram summarizing the results from miRNA Sequencing of human (spindle and cobble) and mepic EV. List at the right hand-side shows highly represented miRNA with cardiomyocyte proliferative potential^56^ that were taken forward and used in AAV synthesis. miRNAs listed in green are exclusive for human spindle (activated) epicardial exosomes.

EVs from a wide range of sources have been described to carry a myriad of cargo, including microRNAs that have previously been implicated as potential mediators of regenerative signals in the context of CVD.^55^ We performed microRNA sequencing of mouse and human primary cobble (epithelial) and spindle (mesenchymal)-derived EVs (data in Supplementary *Table S1*). Enriched microRNAs in epicardial EVs from different sources were consistent, suggesting conservation in microRNA cargo between mouse and human epicardial EVs (*Figure 6*). Interestingly, three microRNAs: miR-99a-5p, miR-146a-5p, and miR-30e-3p, were present in mouse and human spindle (activated) epicardium, but absent from human cobble (inactive) epicardial EVs (*Figure 6*).

### 3.8 miRNAs present in epicardial EVs recapitulate the proliferative response exerted in cardiomyocytes by EV treatment and improve the functional outcome of cryoinjured EHM

We initially tested the effects of individual identified miRNAs on the proliferation of hESC-derived cardiomyocytes. For this purpose, we generated AAV9 vectors expressing the pre-miRNAs for *Homo sapiens* (hsa)-miR-30a and hsa-miR-30e and for a series of other miRNAs derived from the cross-analysis of the epicardial EV miRNA cargo list with a database of human miRNAs, previously identified for their ability to induce proliferation in rat and mouse primary cardiomyocytes, assessed by EdU incorporation.^56^ This published study validates the use of AAV-miRs for testing the ability to induce proliferative responses in hESC-cardiomyocytes, as an initial step to triaging those miRs identified as EV cargoes herein for further *in vivo* testing in future studies.

**Figure 7:**
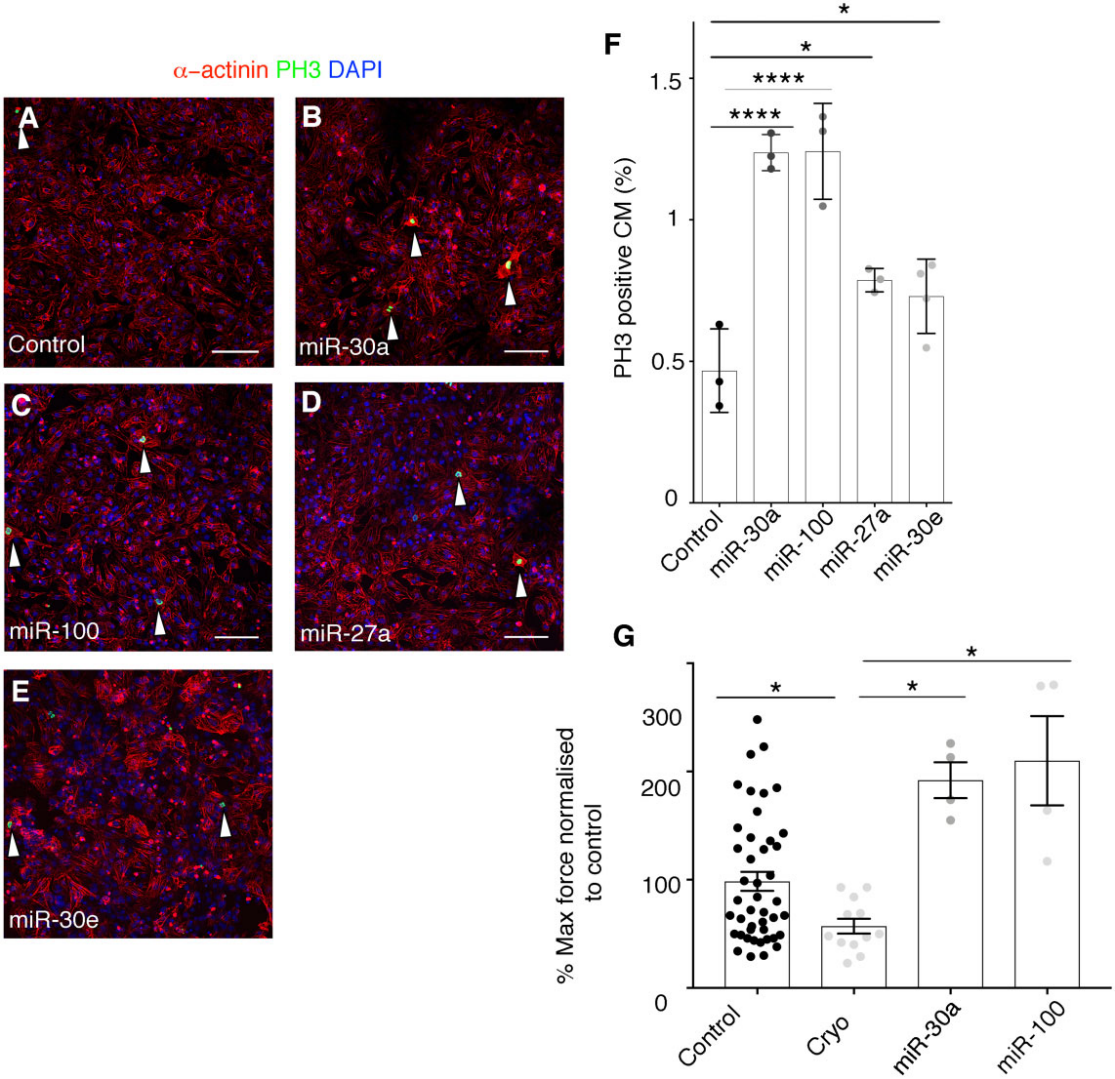
miRNAs present in epicardial EVs partially recapitulate the proliferative ability of EV treatment in cardiomyocytes. (*A*–*F*) Immunostaining of PH3 (green) and α-actinin (red) of cultured mouse neonatal cardiomyocytes, treated with vehicle (*A*), AAV miR-30a (*B*), AAV miR-100 (*C*), AAV miR-27a (*D*), AAV miR-30e (*E*). Arrowheads point at proliferating cardiomyocytes, co-stained with a-actinin and PH3. DAPI (blue) labels cell nuclei. (*F*) Quantification of proliferating cardiomyocytes shown in (*A*–*F*) represented as percentage of cardiomyocytes positive for PH3 staining after treatment with lentiviral particles. (*G*) Quantification of maximum contraction forces of EHM after cryoinjury and treatment with AAV miR-100 and AAV miR-30a at 7 days post-procedure. Data are presented as mean SEM. *n*=3/condition and *n*>3000 cells/condition for (*A*–*F*); and *n*=44 control; *n*=2 cryoinjured; *n*=4 AAVmiR-100; *n*=4 miR30a. *P<0.05, **P <0.01, ***P<0.001, ***P<0.0001. Student’s *t*-test. Scale bars: 50 µm.

These cross-referenced miRNAs included miR-99a, miR-23b, miR-181a, miR-27a, and miR-100.^56^ A significant three-fold increase in PH3^+^ cardiomyocytes was detected 48 h after AAV9-hsa-miR30a and AAV9-hsa-miR100 treatment relative to control (Control 0.47±0.15; miR-30a 1.23±0.04; miR-100 1.24±0.17; miR-27a 0.78±0.04; miR-30e 0.72±0.13, *P*≤0.05 for miR-27a and miR-30e and, *P*≤0.0001 for miR-30a and miR-100) (*Figure 7A–F*). AAV9-hsa-miR27a and AAV9-hsa-miR30e treatment evoked a significant two-fold increase in PH3^+^ cardiomyocytes (*Figure 7D–F*). This data collectively suggest that the combined positive effects on cardiomyocyte proliferation of individual miRNA cargo (miR-30a, miR-100, miR-27a, and miR-30e) may account for the pro-proliferative effects of human epicardial EV treatment. In order to functionally validate the effect of miRNAs present in epicardial EVs in human tissue, we subjected EHMs to cryoinjury and treated them with AAV9-hsa-miR30a and AAV9-hsa-miR100. Analysis of contraction forces a week after injury (when epicardial EVs promoted an improvement in contractility), showed a full recovery of function as compared to cryoinjured EHMs treated with vehicle-alone [Control 98.55±8.86; cryoinjured 57.17±6.98; miR-30a 210±41.26; miR-100 192±16.56, *P*≤0.05 for miR-30a and miR-100) (*Figure 7G*).

## 4. Discussion

This study provides the first account of EVs produced by quiescent versus activated epicardium and an analysis of their functional roles in murine and human models of cardiomyocyte proliferation and heart injury. We describe a pro-proliferative effect of epicardial EVs on cardiomyocytes that implicates signalling from the injury-activated epicardium in regeneration. Other cell types have been shown to secrete EVs that promote modest cardiomyocyte cell cycle activity, although the majority are reported to exert cardioprotective effects.^24^^,^^25^^,^^28^^,^^57^ Many of these prior studies, however, have focused on progenitor or stem cells that are controversial in terms of their origins and precise mechanisms underlying their transient beneficial effects.^58^ The epicardium, due to its known trophic role in the developing heart, anatomical location and well characterized paracrine activity^9^^,^^59^ represents a prime candidate cell-type to reinvoke a proliferative response in cardiomyocytes.

We report efficient uptake of epicardial EVs by cardiomyocytes that promotes an increase in cell cycle activity (*Figure 2* and Supplementary *Figure S2*), mediated via activation of the Akt pathway and partial activation of Hippo and ERK pathways (Supplementary *Figure S3*); all previously implicated in mediating cell cycle activity and proliferation in cardiomyocytes,^45–48^ leading to improvement after MI^60^ and regenerative responses.^46^ Moreover, we show that this effect is also present *in vivo* after injury (Figure 3 and Supplementary *Figures S4 and S5*). In the P1 mouse heart injury model, an increase in proliferation of pre-existing cardiomyocytes has been previously reported as a driving mechanism for regeneration.^13^^,^^52^ Our results suggest that epicardial EVs are able to augment this response in an organ-wide manner (Supplementary *Figures S4 and S5*). Most strikingly, cardiomyocyte proliferation was also increased in EV-treated P7 non-regenerative, infarcted hearts 7 days after injury (*Figure 3* and Supplementary *Figure S5*). This is significant, given the regenerative response has been reported to be lost in mice 1 week after birth (at P7), coinciding with cardiomyocyte cell cycle withdrawal.^13^ Instead, injured P7 hearts adopt the default adult wound healing process of permanent scar formation.^13^ However, despite evoking an early increase in cardiomyocyte proliferation in the EV-treated P7 infarcted hearts, we were unable to detect evidence of longer-term benefit; with no effect on scarring, vascularization or myocardial disarray to indicate more optimal repair or reduced pathological remodelling (Supplementary *Figure S6*). Regeneration is a complex process, in which different cell types contribute to tissue restoration at the expense of scarring and remodelling via simultaneous and synergistic roles. Thus, it is possible that even though epicardial EVs promote an increase in cardiomyocyte cell cycle activity in non-regenerative postnatal hearts, it is not enough to promote regeneration. This contrasts with the view that places cardiomyocyte proliferation as the major or exclusive driver of regeneration in both neonatal mice and adult zebrafish,^13^^,^^15^^,^^31^^,^^51^^,^^61^^,^^62^ but reflects the fact that other factors, such as collateral blood vessel formation,^63^ macrophages,^52^ and extracellular matrix,^32^^,^^64^ are also important contributors to heart regeneration. One major limitation of our study is the lack of functional *in vivo* data to assess whether the increase in cardiomyocyte cell cycle activity, mediated by epicardial EV treatment after injury, in a non-regenerative model is able to improve heart function regardless of a lack of full regeneration. This limitation can be addressed by adaptation of functional imaging to the neonatal model in future studies.

We also describe for the first time the release of EVs by human epicardial cells, derived from RAA patient biopsies and hESC-derived epicardial cells (Figure 4 and Supplementary *Figure S7*). Reports of EVs isolated from the pericardial fluid of patients undergoing aortic valve surgery^21^ suggested putative EV secretion by epicardial cells; however, the cellular source was not identified. In this study, human epicardial-derived EVs induced a significant proliferative response in cardiomyocytes that mimicked that of mouse EVs. To analyse human EV effects related to myocardial injury, we established cryoinjury of three-dimensional EHM^41^ constructs.^65^^,^^66^ We ensured injury to a localized site by repeat application of the cryoprobe, which resulted in irreversible structural and functional damage at days 1–3 post-injury. Epicardial EV treatment of injured EHMs invoked significant cardiomyocyte proliferation, which resulted in functional recovery 7 days post-injury (*Figure 5* and Supplementary *Figure S9*). The transfer of EV cargo is predicted to be a rapid event and, therefore, the delay in improved outcome, as observed at 7 days, likely reflects the need to invoke sufficient cardiomyocyte proliferation, subsequent maturation and functional integration within the injured tissue prior to impacting positively on contractile function.

EVs mediate their biological effects *via* specific cargoes, and have been reported to transport proteins, nucleic acids and lipids.^67^^,^^68^ Their ability to transport microRNAs in particular has been a major focus regarding mechanisms of cell–cell signalling^28^^,^^57^^,^^69^ We identified miRNA cargo in epicardial EVs by deep RNA-sequencing and tested whether miRNAs residing in mouse and human EVs might be responsible for inducing cardiomyocyte proliferation and the pro-regenerative response observed in the neonatal mouse and human EHM models. Interestingly, we identified a series of miRNAs (*Figure 6*) (miR-99a-5p, miR-30e-3p, miR-30a-5p, miR-21-5p, miR-23b-3p, miR-181a-5p, miR-27a-3p, miR-100-5p, and miR-146a-5p) common to both epicardial sources, suggesting conserved function. Moreover, we observed a shift in miRNA content when comparing EVs from activated (spindle; mesenchymal) and inactive (cobble-like; epithelial) epicardial cells that induced different levels of responses on target cardiomyocytes. Specific miRNAs were also unique to spindle epicardial cell EVs (including miR-30e), which suggests a specific role in promoting a more potent proliferative response once the epicardial cells undergo EMT after activation.^5^ From our data, no single miRNA exerted a proliferative response to the same extent as EV treatment; albeit individually they were able to increase proliferation in human ES-derived cardiomyocytes (*Figure 7*), suggesting a combinatorial and synergistic role for the cohort of identified cargo miRNAs. In addition, other putative unexplored epicardial EV cargoes, such as alternate nucleic acids (mRNA and lncRNA) and protein might also contribute to the overall EV-enhanced proliferative effect on cardiomyocytes. Interestingly, two of the miRNAs (miR-30 and miR-100) were also able to elicit a functional improvement in injured EHMs, comparable to EV treatment (*Figure 7G*) suggesting more potent effects within human myocardial tissue.

Some of the most abundant miRNA species in epicardial EVs have previously been implicated therapeutically in the context of CVD. miR-21 secreted by bone marrow-derived cells has been reported to enhance tissue contractility in mature ischaemic adult cardiomyocytes.^70^ Moreover, miR-21 has been shown to promote a cardioprotective effect via regulation of cell death and survival pathways, contributing to reduced myocardial damage.^71^ Other highly represented miRNA species in epicardial EVs have not previously implicated in CVD, but share common downstream targets that could mediate cardiomyocyte proliferation: such as Tor (miR-30a, miR-100, and miR-30e)^72^ and Notch (miR- 21 and miR-30a^73^). They have also been implicated in metabolic regulation and autophagic responses (miR-30a^74^^,^^75^ and miR-27a^72^), which can lead to increased proliferation and increased protection against cellular damage.^76^

In conclusion, this study is the first to implicate EV release as a mediator of epicardium-derived paracrine signalling. Epicardial EVs from both murine and human sources have the potential to invoke cardiomyocyte cell cycle re-entry, both *in vitro* and *in vivo*, mediated by conserved miRNA cargo, which results in improved outcome in human heart injury models. Moreover, activated epicardial cells having undergone EMT present with a different EV signature as compared to when residing within the epicardium proper, with altered miRNA cargo that may increase myocardial proliferation. The focus herein has been on invoking cardiomyocyte proliferation to potentially extend the regenerative window in the neonatal mouse model, and in a more developmental model of EHM following injury; further studies are required to extrapolate the findings to the adult setting post-MI. Thereafter, understanding and targeting EV release from activated epicardium may represent a therapeutic opportunity for treating patients following acute MI.

## Supplementary material

Supplementary material is available at *Cardiovascular Research* online.

## Authors’ contributions

C.V.d.C. and N.Y.L.: Study conception and design; Acquisition of data; Analysis/interpretation of data; Drafted manuscript; Critical revision. M.G.-R.: Performed MI surgery. M.M.: Acquisition of data; Analysis/interpretation of data. L.B.: Generated and provided AAV vectors. N.V.: Provided hRAA. T.K.: Isolated EVs. M.G.: Generated and provided AAV vectors. G.S.: EV miRNA isolation and sequencing. W.-H.Z.: Analysis/interpretation of data; Critical revision. P.R.R.: Study conception and design; Analysis/interpretation of data; Drafted manuscript; Critical revision.

### Acknowledgements

We acknowledge the assistance of Prof. Dr Ingo Kutschka for the provision of primary atrial muscle biopsies and Prof. Dr Sonja Schrepfer (Hamburg, Germany) for providing the hES2-LUC^+^ stem cell line. We acknowledge UMG sequencing facility NIG for miRNA content analyses on EV cargo. We thank the Micron Oxford Advanced Bioimaging Unit for access to and training in the use of confocal microscopy.

**Conflict of interest**: W.-H.Z. is founder and advisor of Repairon GmbH and myriamed GmbH. P.R.R. is cofounder of and equity holder in OxStem Cardio. All other authors have no conflict of interest to declare.

### Funding

This work was funded by the British Heart Foundation (BHF Chair Award to P.R.R.: CH/11/1/28798) and the Leducq Transatlantic Network (#14 CVD 04).

## Data availability

The data underlying this article will be shared on reasonable request to the corresponding author. The data underlying this article corresponding to miRNA sequencing are available in Gene Expression Omnibus.

## Supplementary Material

cvab054_Supplementary_Data

## Notes

Translational perspective Coronary artery disease leading to MI (‘heart attack’) is a major cause of death worldwide. After a heart attack, the myocardium is unable to regenerate; therefore, the heart remains permanently scarred and unable to carry out its function, which eventually leads to heart failure. Current therapies are palliative, aimed at unburdening the failing heart, or heart transplantation. Reprogramming adult cardiomyocytes to a proliferative state, via the use of non-inmunogenic EVs is a potential therapeutic approach to restore lost heart muscle, resolve scarring and prevent heart failure progression.
