# Supplementary material for: Regenerative potential of epicardium-derived extracellular vesicles mediated by conserved miRNA transfer
Source: Cardiovasc Res. 2021 Feb 18;118(2):597–611. doi: 10.1093/cvr/cvab054 (PMC8803084; doi:10.1093/cvr/cvab054)
Supplement: cvab054_Supplementary_Data [file cvab054_supplementary_data.zip › cvab054-suppl_data/Supplementary material_WB_Resub mission.pdf]

Supplementary Data

Title: Regenerative potential of epicardium-derived extracellular vesicles mediated by conserved miRNA transfer

Cristina Villa del Campo[1], Norman Y. Liaw[2,3], Mala Gunadasa-Rohling[1], Moritz Matthaei[2], Luca Braga[4,5], Tahnee Kennedy[1],Gabriela Salinas[6], Niels Voigt[2,3], Mauro Giacca[4,5], Wolfram-Hubertus Zimmermann[2,3], Paul R. Riley[1,*]

[1] Department of Physiology, Anatomy and Genetics, British Heart Foundation, Oxbridge Centre of Regenerative Medicine, University of Oxford, UK.

[2] Institute of Pharmacology and Toxicology, University Medical Center Göttingen, Germany.

[3] DZHK (German Centre for Cardiovascular Research), partner site Göttingen, Germany.

[4] Molecular Medicine Laboratory, International Centre for Genetic Engineering and Biotechnology (ICGEB), Trieste, Italy.

[5] School of Cardiovascular Medicine & Sciences, British Heart Foundation Centre, King's College London, UK.

[6] NGS- Integrative Genomics Core Unit (NIG) Institute of Human Genetics; University Medical Centre Göttingen (UMG), Germany

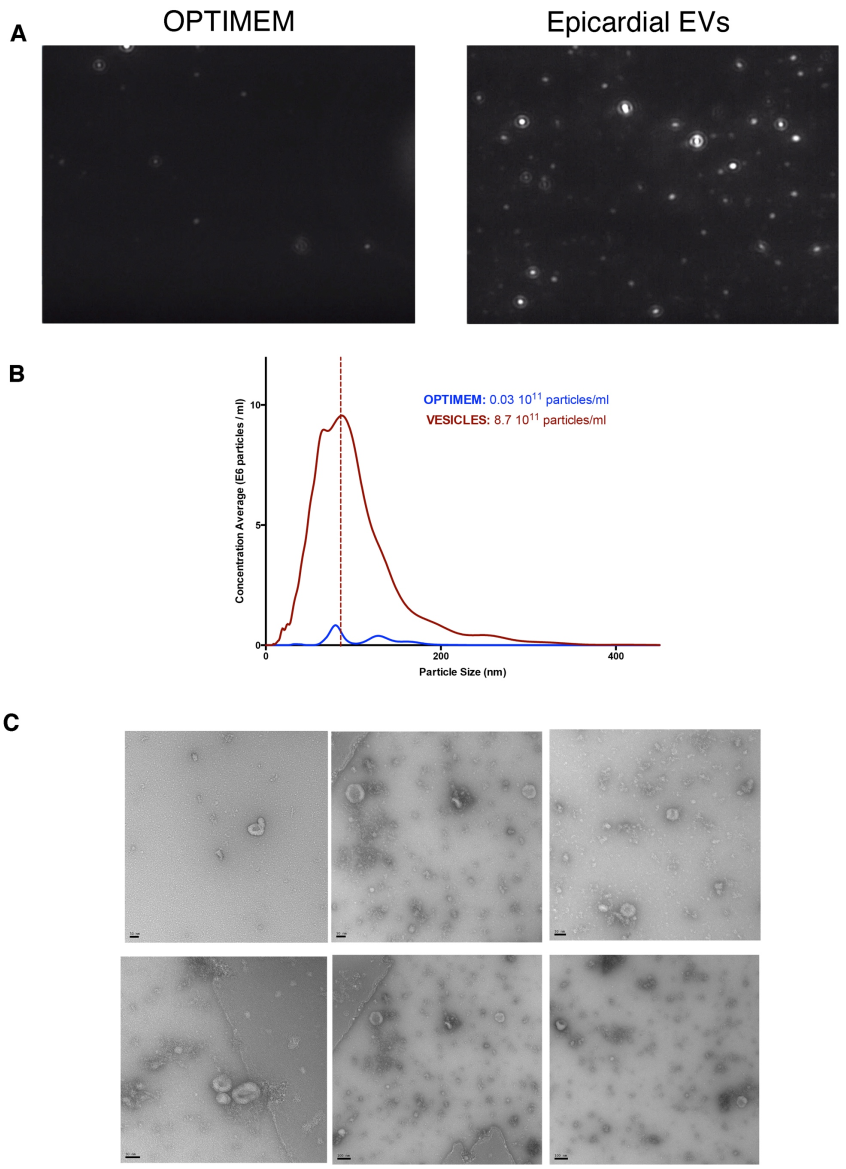

Supplementary Figure 1

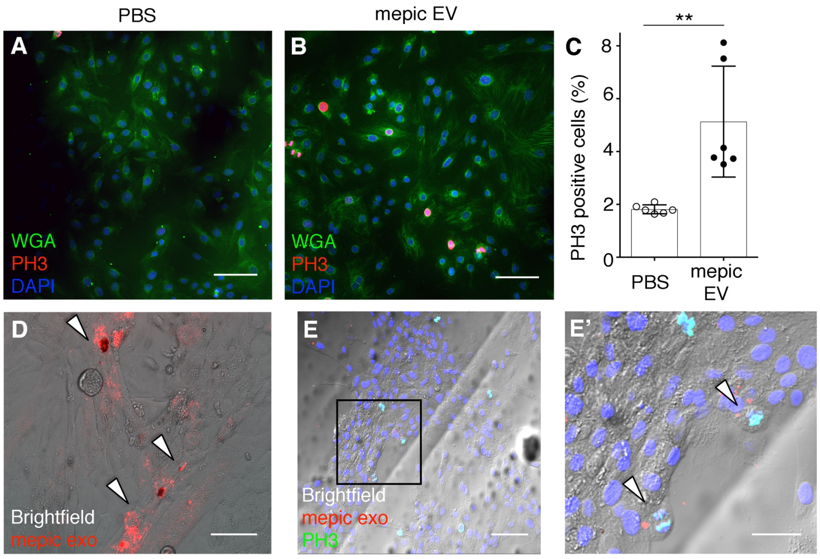

Supplementary Figure 2

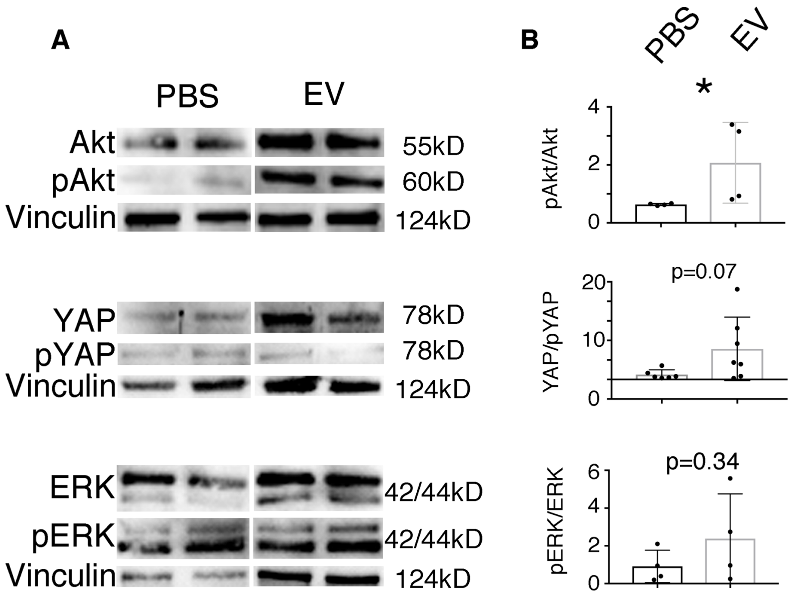

Supplementary Figure 3

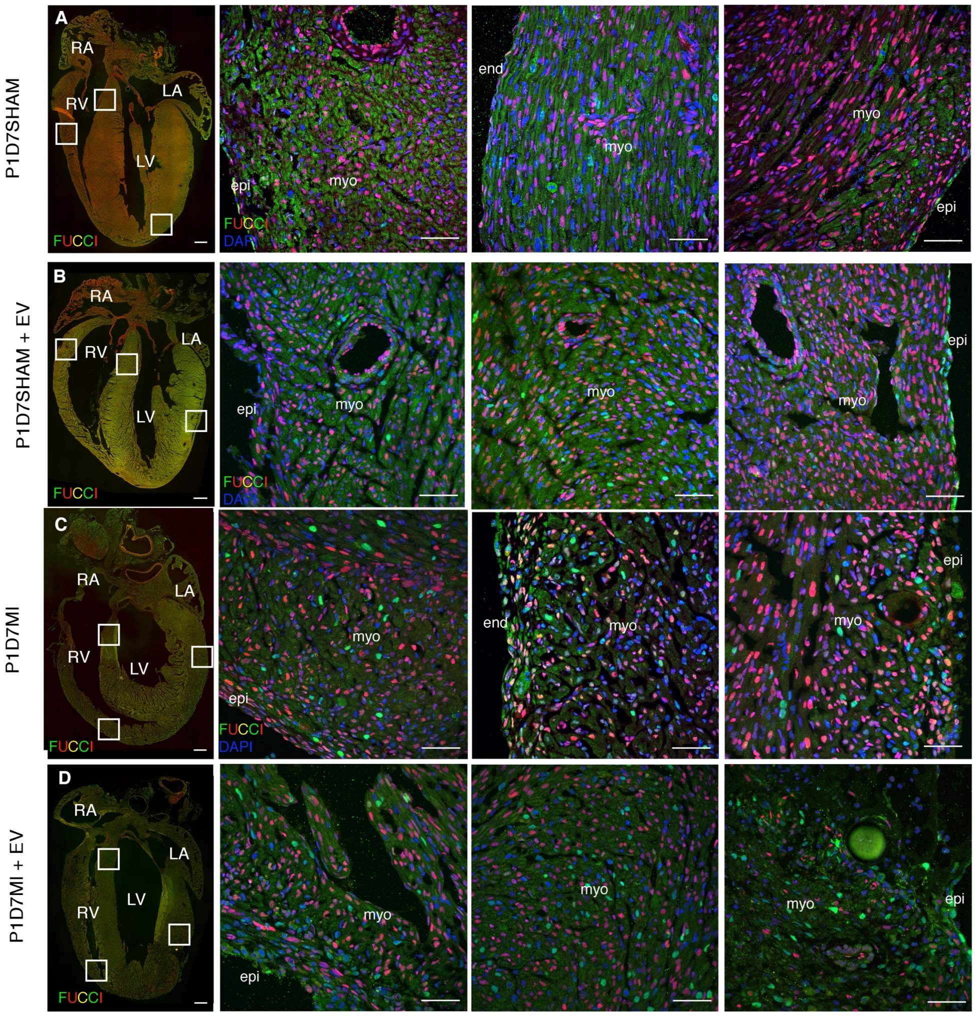

Supplementary Figure 4

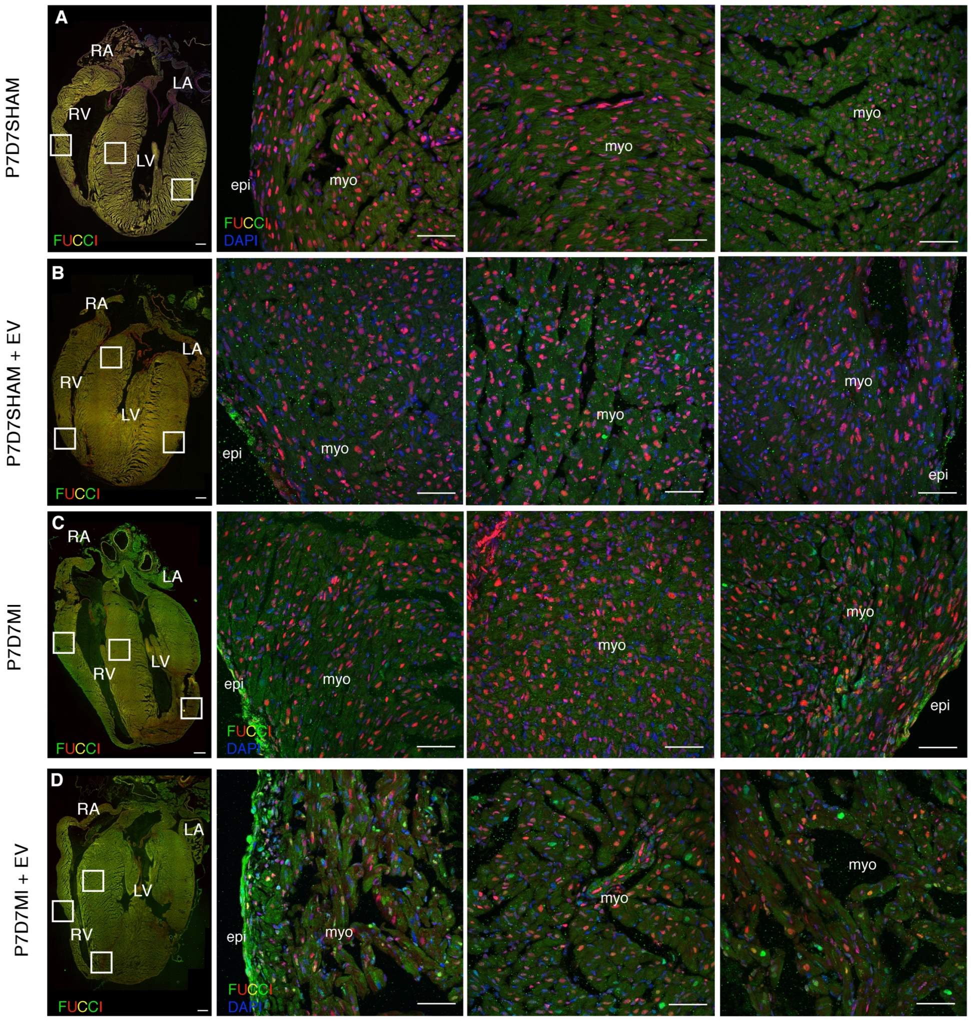

Supplementary Figure 5

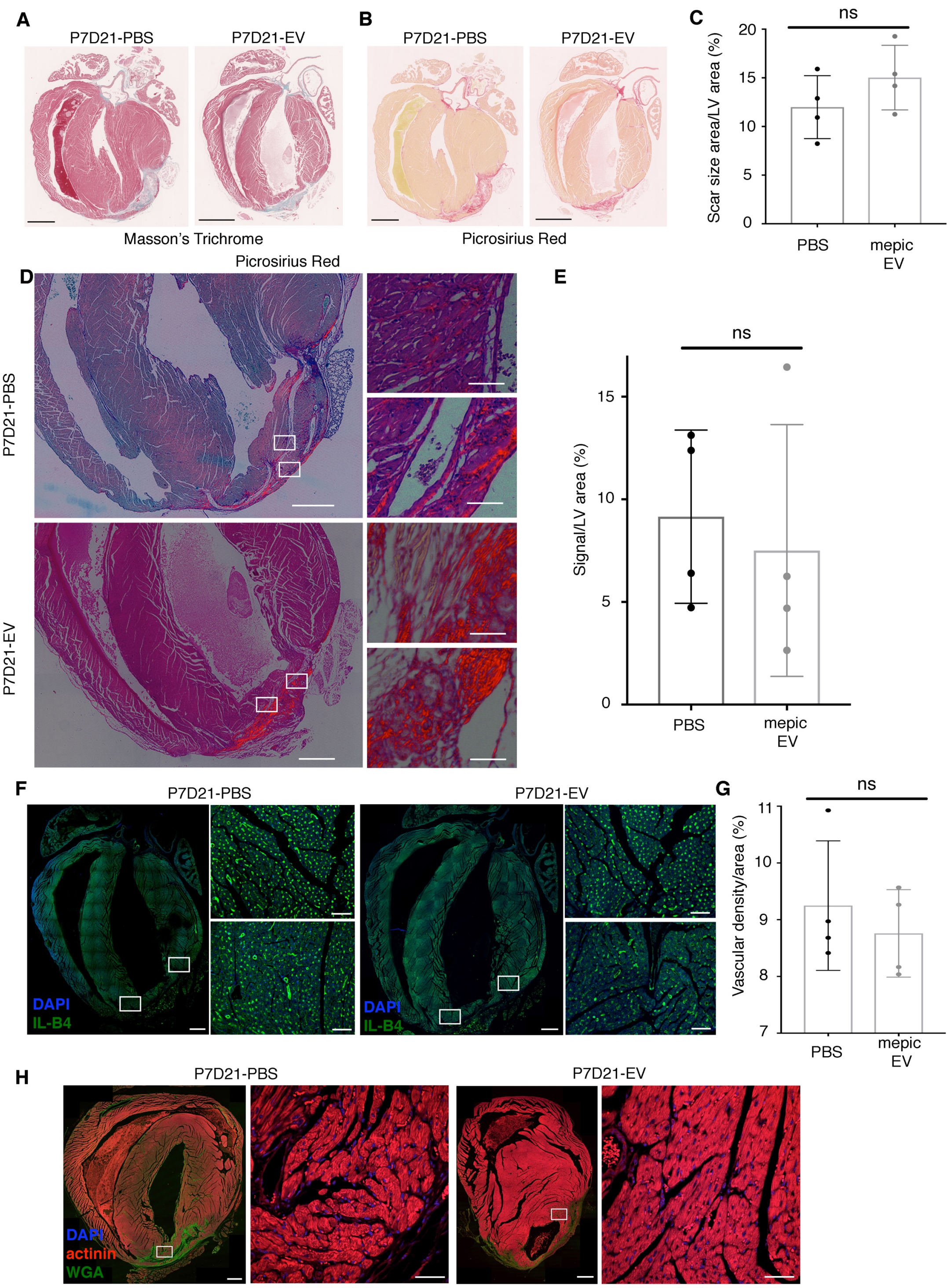

Supplementary Figure 6

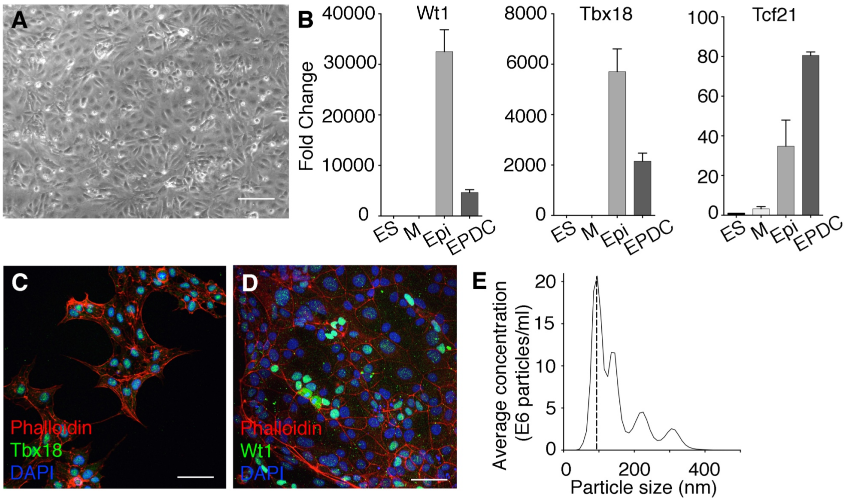

Supplementary Figure 7

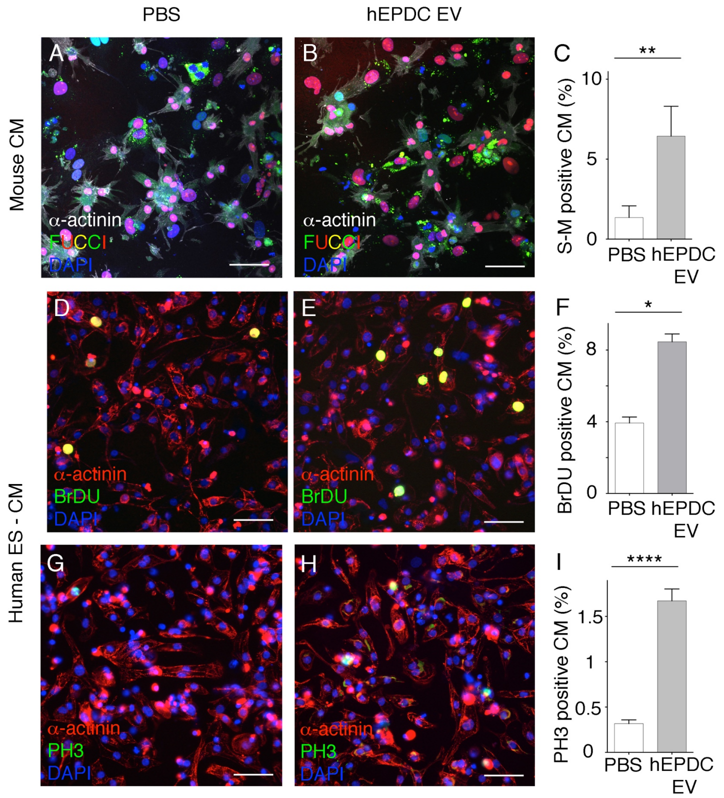

Supplementary Figure 8

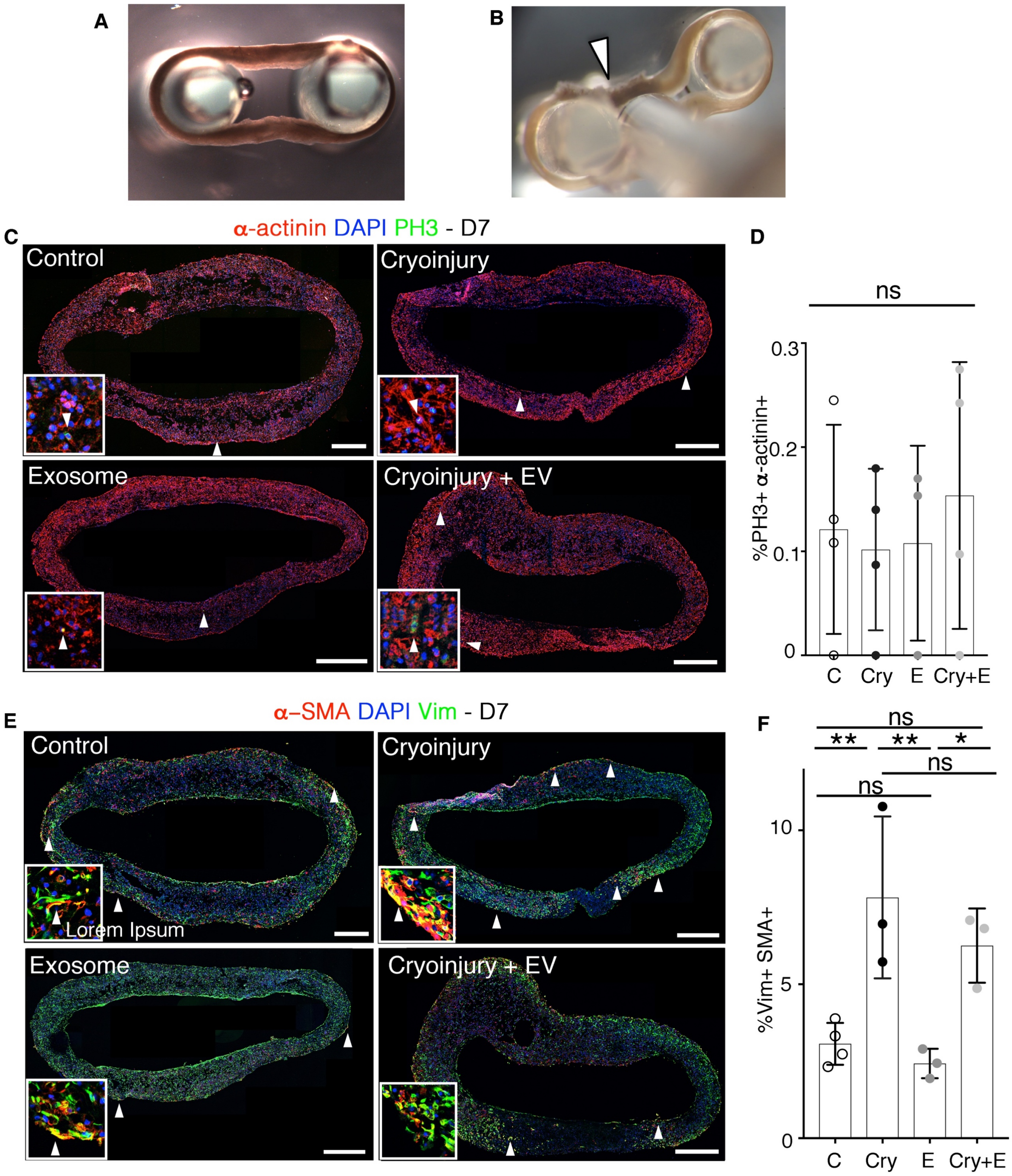

Supplementary Figure 9

Figure S1. Epicardial EVs after isolation as detected by TEM and NTA

A. OPTIMEM (left) and isolated epicardial EVs (right) video stillshots obtained from NTA detection and further used for concentration and particle size quantification. **B.** OPTIMEM (blue) and epicardial EV (red) profile of particle size and concentration as obtained from NTA analysis. Mode of EV size is 90nm. **C.** Representative images obtained from TEM of counterstained epicardial EV. Scale bar: 50nm in upper panels and bottom left panel and 100nm in bottom centre and right panels. Note the cup-shaped morphology and EV size (between 50 and 100nm).

Figure S2. Mouse epicardial EV treatment increases cell cycle activity in H9C2 cells

A, B. Confocal images showing PH3 (red) and WGA (green) in H9C2 cells treated with vehicle (**A**) or mouse epicardial (mepic) EVs (**B**). DAPI (blue) labels cell nuclei. **C.** Quantification of the percentage of PH3 positive cells after treatment with epicardial exosomes or vehicle. **D**. Brightfield and PKH26 (red) image of cultured cells 24 hours after treatment with PKH26 (red staining) labelled mouse epicardial EVs. Arrowheads point to uptaken labelled EVs. **E.** H9C2 cells culture treated with mouse epicardial EVs labelled with PKH26 (red) and stained for PH3 (green). Cells are seen in brightfield and nuclei are labelled with DAPI (blue). Boxed area is magnified in **E'**. Arrowheads point to PH3 positive H9C2 cells that have uptaken labelled EVs. Note correlation between EV uptake and proliferation. Data are presented as mean SEM. n= 6/condition. *p<0.05 **p<0.01 ***p<0.001 by Mann-Whitney statistical test. Scale bars: 100μm (A, B, E), 50μm (D, E').

Figure S3. Activated downstream pathways mediating cell cycle activity responses upon epicardial EV treatment

A. Western blot analysis of mouse primary cardiomyocytes for Akt, pAkt, YAP, pYAP, ERK and pERK, with Vinculin used as a loading control. Lanes on the left display PBS-treated cardiomyocyte and lanes on right, EV-treated cardiomyocytes. **B.** Quantification of the ratio of pAkt/Akt (upper); YAP/pYAP (middle) and pERK/ERK (bottom) in PBS and EV-treated neonatal primary cardiomyocytes. Data are presented as mean SEM. n=4 for pAKT/AKT PBS, n=4 for pAKT/AKT EV; n=6 for YAP/pYAP PBS; n=7 for YAP/pYAP EV-treated; n=4 for pERK/ERK PBS, n=4 pERK/ERK PBS. N-numbers correspond to biological replicates. *p<0.05, by Student t-test.

Figure S4. FUCCI reporter labelling in the P1 regenerative MI model with epicardial EV treatment.

A-D. FUCCI cell cycle reporter (red and green) and DAPI to label cell nuclei are shown in P8 heart sections collected 7 days after sham or MI procedure followed by epicardial EV or vehicle injection performed at P1. **A**: Sham, **B**: Sham with administration of EVs, **C**: MI; **D**: MI with administration of EVs. Left panels show lower power images of the whole heart section and representative boxed areas are shown in a higher power in the middle and right panels. Scale bars: 200μm left panels, 50μm enlarged boxed areas. LV: left ventricle, LA: left atria, RV: right ventricle, RA: right atria, epi: epicardium, myo: myocardium, end: endocardium

Figure S5. FUCCI reporter labelling in the P7 non-regenerative MI model with epicardial EV treatment.

A-D. FUCCI cell cycle reporter (red and green) and DAPI to label cell nuclei are shown in P14 heart sections collected 7 days after sham or MI procedure followed by EV or vehicle injection performed at P7. **A**: Sham, **B**: Sham with administration of EVs, **C**: MI; **D**: MI with administration of EVs. Left panels show lower power images of whole heart sections and representative boxed areas are shown in a higher power in the middle and right panels. Scale bars: 200μm left panels, 50μm enlarged boxed areas. LV: left ventricle, LA: left atria, RV: right ventricle, RA: right atria, epi: epicardium, myo: myocardium, end: endocardium

Figure S6. Mouse epicardial exosome injection in P7 injured hearts does not promote long-term regenerative responses.
A. Brightfield histological images stained for Masson's Trichrome are shown in P28 heart sections collected 21 days after MI procedure followed by exosome (EV) or vehicle (PBS) (left) injection performed at P7. **B**. Brightfield histological images stained for Picrosirius Red are shown in P28 heart sections collected 21 days after MI procedure followed by exosome (EV) or vehicle (PBS)(left) injection **C**. Quantification of the percentage of the scarred area (by blue staining in Masson's Trichrome) in the left ventricle in percentage. **D**. Polarised light microscopic images of Picrosirius Red staining in P28 heart sections collected 21 days after MI procedure followed by EV (EV) or vehicle (PBS) (left) injection performed at P7. Left panels show lower power images of whole heart sections and representative boxed areas are shown in a higher power in the right panels. **E**. Quantification of the percentage of the stained area as seen by polarised light in the left ventricle in percentage. **F.** Microscopic images showing isolectin B4 (IL-B4) (green) and DAPI (blue) staining in P28 heart sections collected 21 days after MI procedure followed by EVs (EV) or vehicle (PBS) (left) injection performed at P7. Left panels show lower power images of whole heart sections and representative boxed areas are shown in a higher power in the right panels. **G.** Quantification of the vascular density as percentage of the area of IL-4 per tissue area in the injured region. **H.** Microscopic images showing wheat germ agglutinin (WGA) (green), α-actinin (red) and DAPI (blue) staining in P28 heart sections collected 21 days after MI procedure followed by EV (EV) or vehicle (PBS) (left) injection performed at P7. Left panels show lower power images of whole heart sections and representative boxed areas are shown in a higher power in the right panels.
Scale bars: 200μm in A, B and D. 500μm in F and H. 50μm enlarged boxed areas.
Data are presented as mean SEM, Mann-Whitney statistical test. n= 4, PBS injected; n= 4, EV injected (5 sections were analysed per sample).

Figure S7. Epicardial cells derived from human stem cells secrete exosome-like extracellular vesicles.
A. Microscope image of epicardial cells derived from cultures of H9 hES in vitro. **B**. *Tbx18* and *Wt1* expression analysis by qRT-PCR showing comparison of ES-derived epicardial cells [11], ES, mesodermal (M) cells and human EPDCs derived from patient RAA biopsies. **C.** Expression of Tbx18 as epicardial marker in hES-derived epicardial cells. **D.** Expression of Wt1 as epicardial marker in hES-derived epicardial cells. Nuclei are labelled with DAPI (blue). **E.** Nanosight profile of EV isolated from hES-derived epicardial cells, showing a mode of particle size of 95nm. Data are presented as mean SEM in **B** and frequency of EVs in a given size in **E**. n= 4/condition. *p<0.05 **p<0.01 ***p<0.001 Mann-Whitney statistical test. Scale bars: 100μm (A), 50μm (C, D).

Figure S8. Human EVs derived from epicardial cells isolated from RAA biopsies induce cell cycle activity in mouse neonatal cardiomyocytes and human ES-derived CM in 2D culture.

A,B. FUCCI cell cycle reporter (red and green) cultured mouse neonatal cardiomyocytes treated with vehicle (**A**) or human epicardial EVs (**B**). a-actinin is shown in grey and DAPI (blue) labels cell nuclei. **C**. Quantification of cardiomyocytes' cell cycle reported by FUCCI and co-staining with a-actinin. Graph shows the percentage of a-actinin positive cells that display FUCCI Venus reporter. **D, E.** Immunostaining for BrDU (green) and a-actinin (red) of human ES-derived cardiomyocytes, treated with vehicle (**D**) or human epicardial EVs (**E**). **F.** Quantification of proportion of cardiomyocytes (α -actinin positive) positive for BrdU in both vehicle and exosome-treated cultures. **C.** Quantification of proportion of human ES-derived cardiomyocytes (α-actinin positive) positive for PH3 in both vehicle and exosome-treated cultures. **C, D.** Immunostaining for BrdU (green) and α -actinin (red) of cultured human ES-derived cardiomyocytes, treated with vehicle (**D**) or human epicardial EVs (**E**). **F.** Quantification of proportion of human ES-cardiomyocytes (α -actinin positive) positive for BrdU in both vehicle and EV-treated cultures. **G, H.** Immunostaining of PH3 (green) and α -actinin (red) of cultured human ES-derived cardiomyocytes, treated with vehicle (**G**) or human epicardial EVs (**h**). Quantification of proportion of cardiomyocytes (α -actinin positive) positive for PH3 in both vehicle and EV-treated cultures. Data are presented as mean SEM. n=4 FUCCI, Control; n= 4 FUCCI, EVs n= 4 PH3, Control; n= 4 PH3, EVs; n= 3 BrDU, Control; n= 3 BrdU, EVs. *p<0.05 **p<0.01 ***p<0.001 Scale bars: 50μm

Figure S9. Human engineered myocardium response 7 days after cryoinjury and treatment with human epicardial EVs

A. Brightfield scope image of a EHM prior to injury in the passive stretchers. **B**. Stillshot captured from video after cryoinjury procedure in EHM. Arrowhead points to the injured area in the EHM arm. **C.** α -actinin (red) and PH3 (green) confocal images of EHMs at day 7 after procedure for the four experimental groups. Arrowheads point to PH3 positive cardiomyocytes. Boxed areas show magnification of representative cells displaying co-staining of PH3 and actinin. **D.** Quantification of the percentage of cardiomyocytes expressing PH3 in EHMs (C: control, Cry: cryoinjured, E: EVs, Cry+E: cryoinjured treated with EVs). **E**. α -SMA (red) and vimentin (green) staining of EHMs 7 days after procedure. Arrowheads point to α -SMA positive fibroblasts (vimentin positive cells). Boxed areas show magnification of representative cells displaying co-staining of vimentin and α -SMA. **F**. Quantification of the percentage of vimentin cells co-expressing α -SMA in EHMs (C: control, Cry: cryoinjured, E: EVs, Cry+E: cryoinjured treated with EVs). Data are presented as mean SEM. n= 4 Control d3; n= 3 Exosome treated d3; n= 4 Cryoinjured d3; n= 4 cryoinjured EV treated d3; n= 4 Control d3; n= 3 EV treated d3; n= 4 Cryoinjured d3; n= 4 cryoinjured EV treated d3. *p<0.05 **p<0.01 ***p<0.001 Mann-Whitney statistical test. Scale bars: 200μm.

Video S1. Video showing the procedure for inducing a cryoinjury in engineered myocardial constructs.

Table S1. miRNA counts for human, mouse spindle and mouse cobble epicardial-derived exosomes

Table S2. Antibodies used

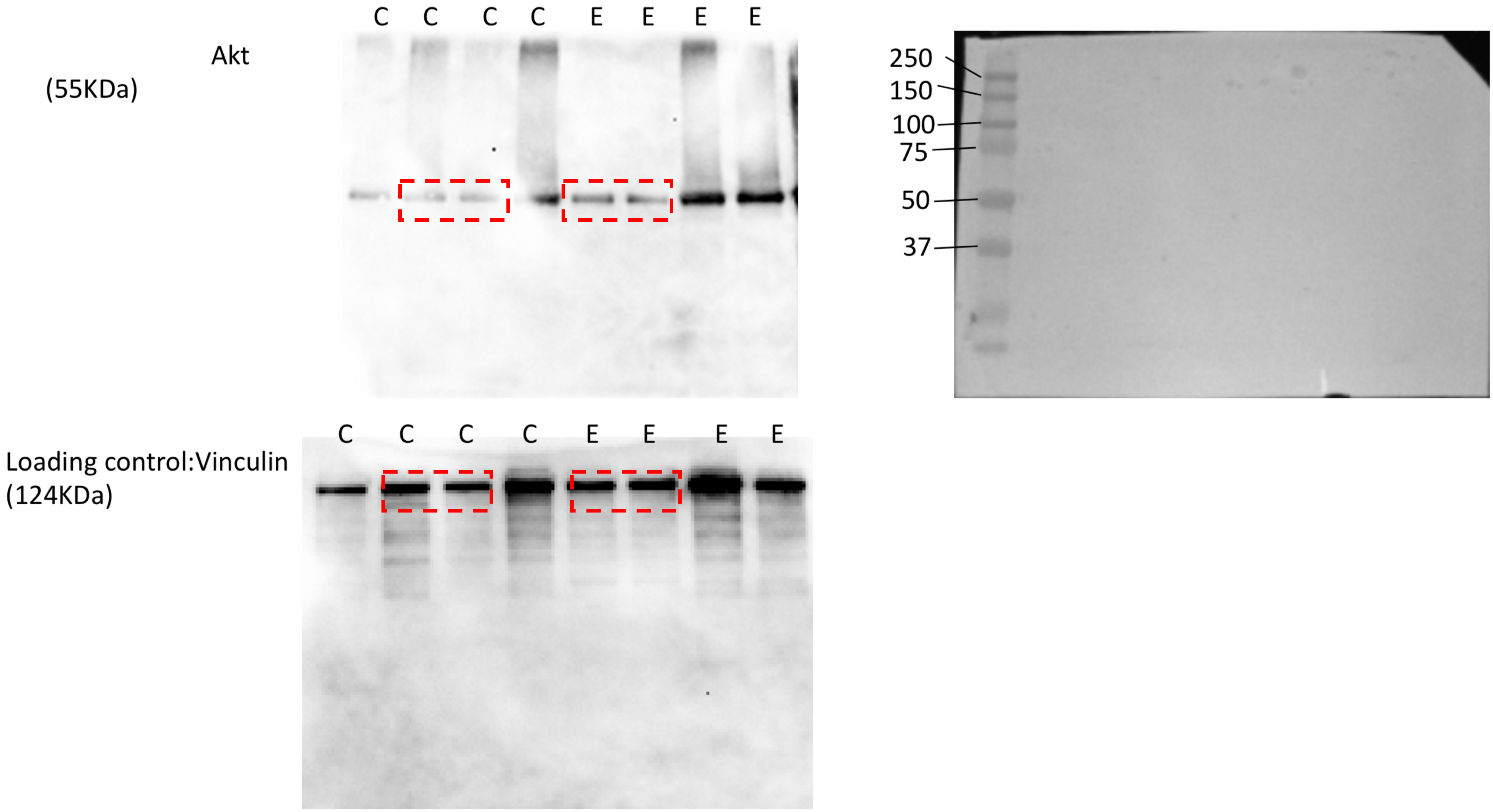

Akt
(55KDa)
C C C C E E E E
250
150
100
75
50
37
Loading control:Vinculin
(124KDa)
C C C C E E E E

pAkt

(60KDa)

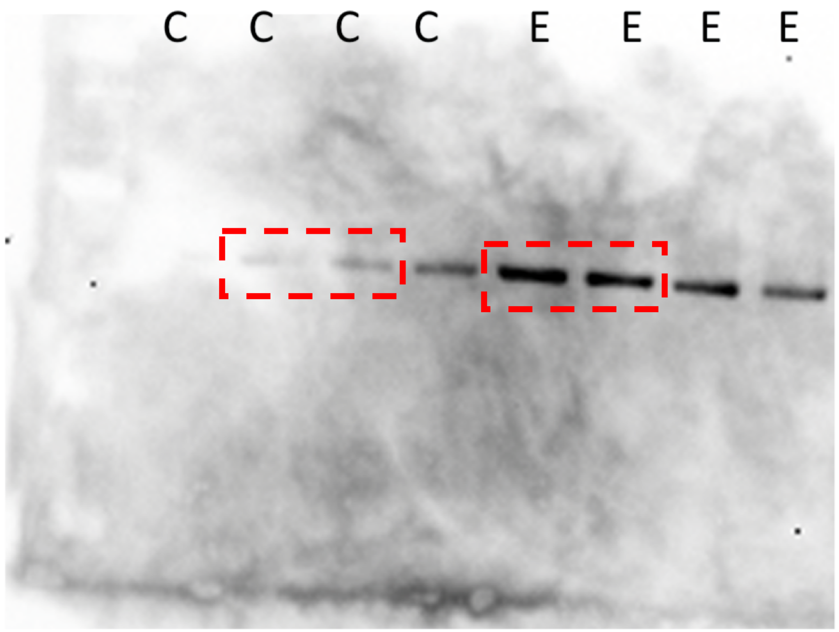

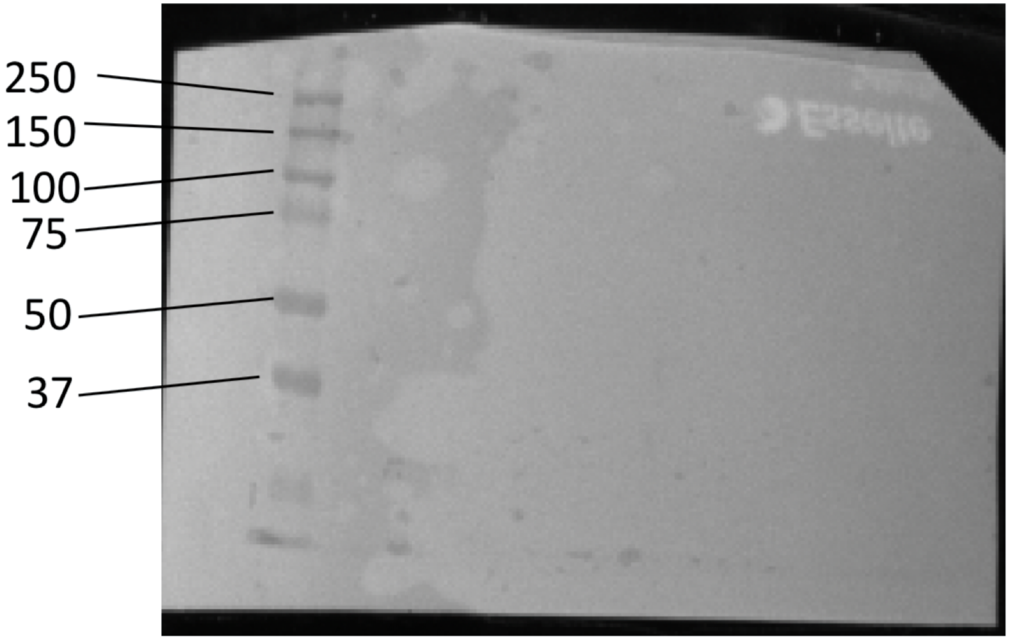

Vinculin (124kDa)
pAkt (60KDa)

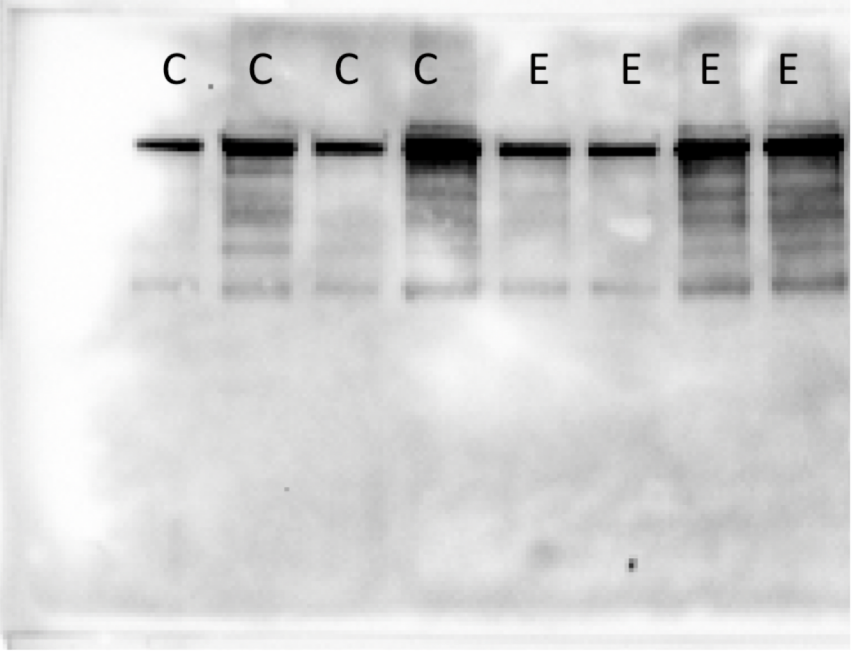

ERK (42 and 44KDa)

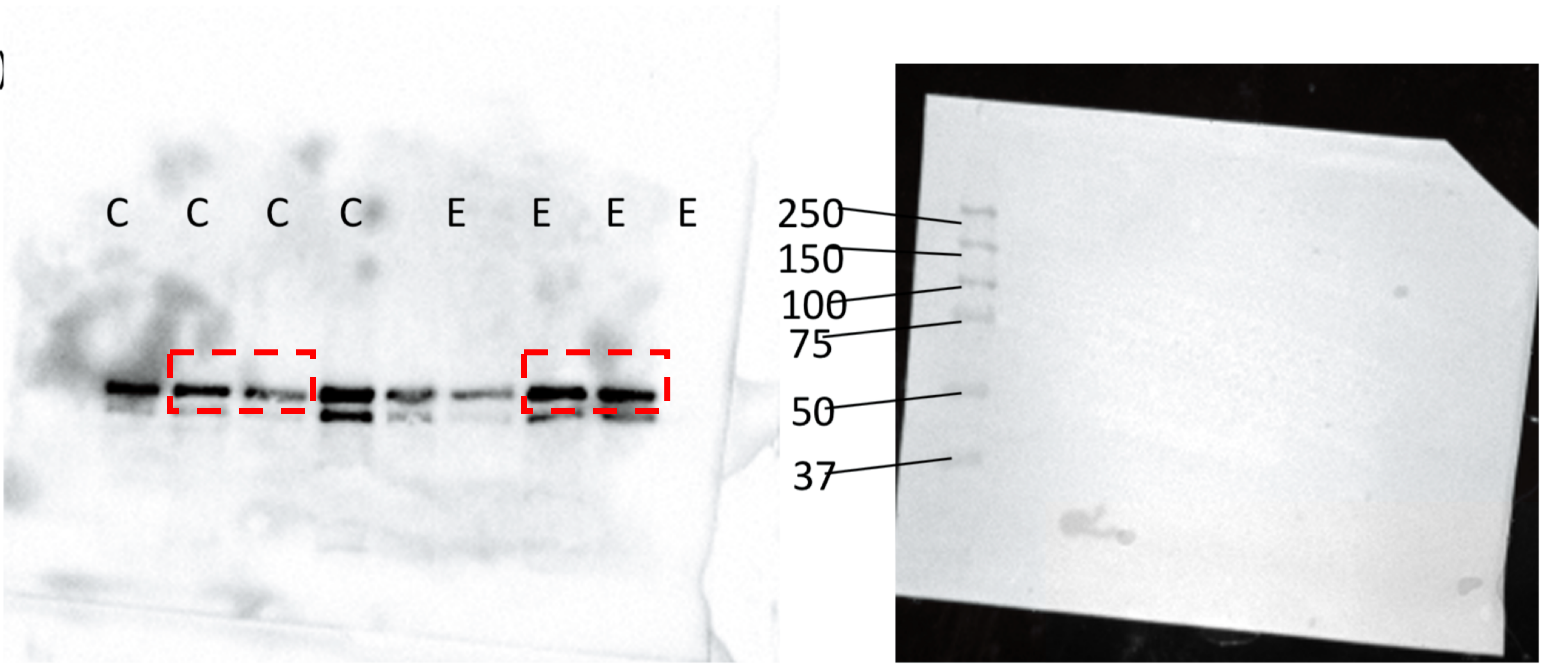

Vinculin (126KDa)
ERK (42 and 44KDa)

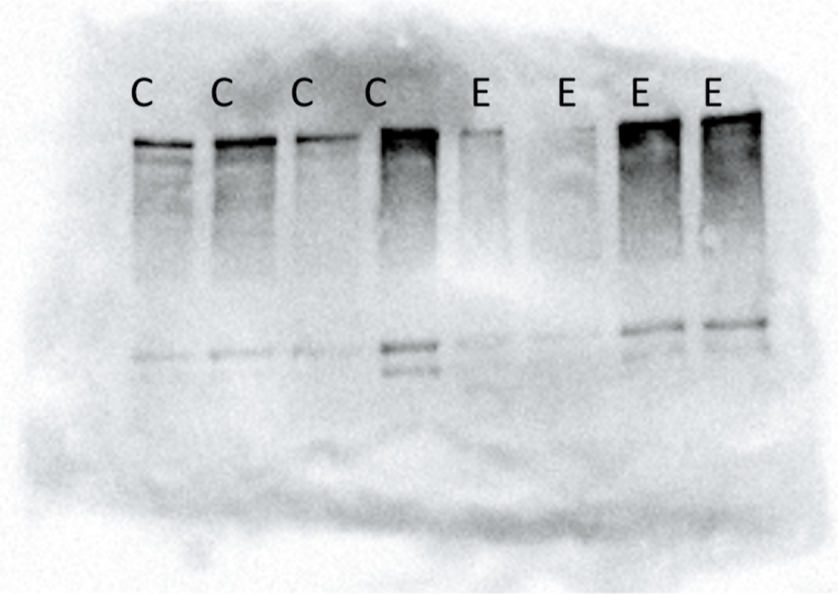

pERK (42 and 44KDa)

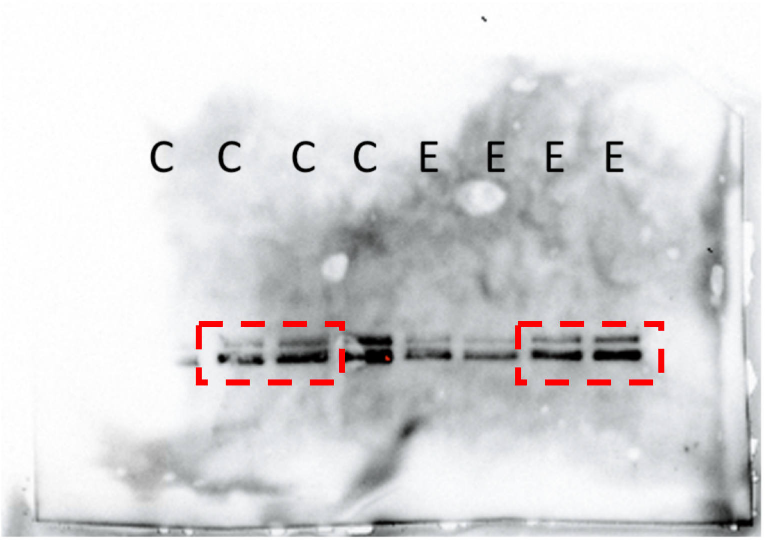

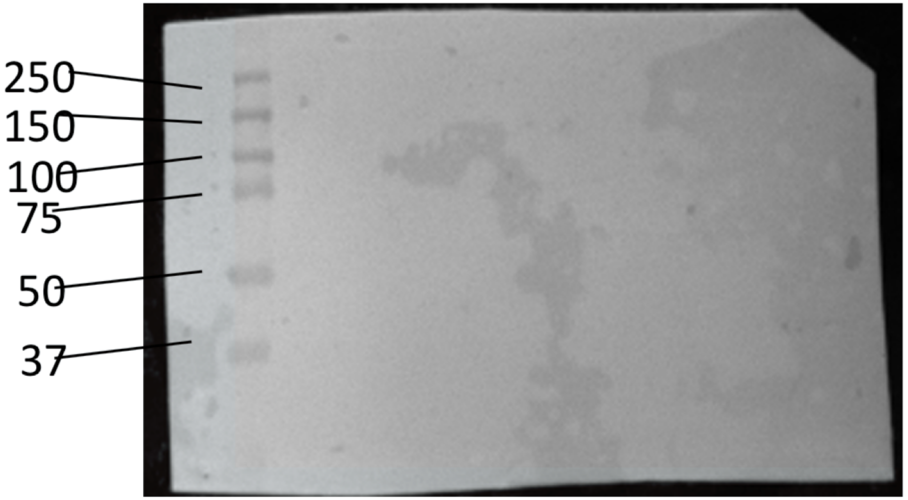

Vinculin (126KDa)
pERK (42 and 44KDa)

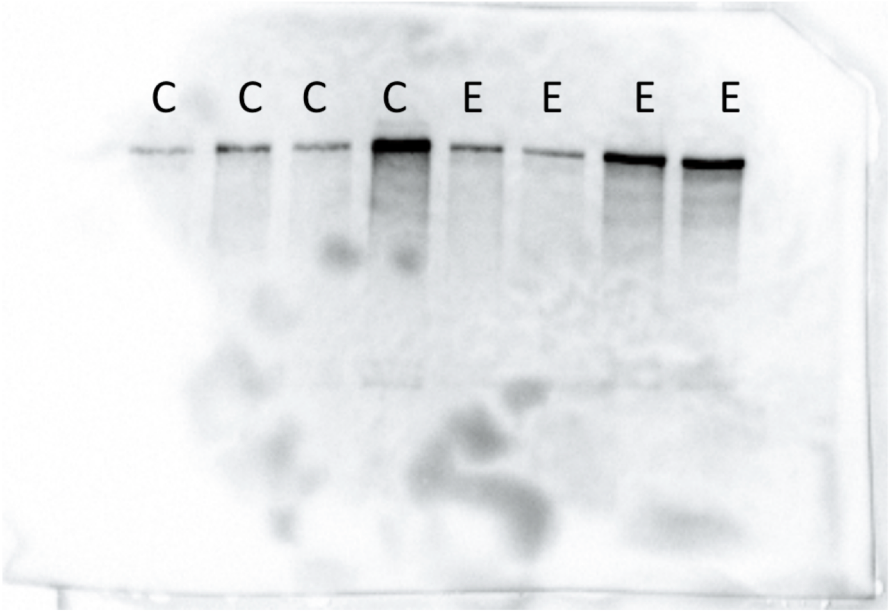

YAP (65-78KDa)

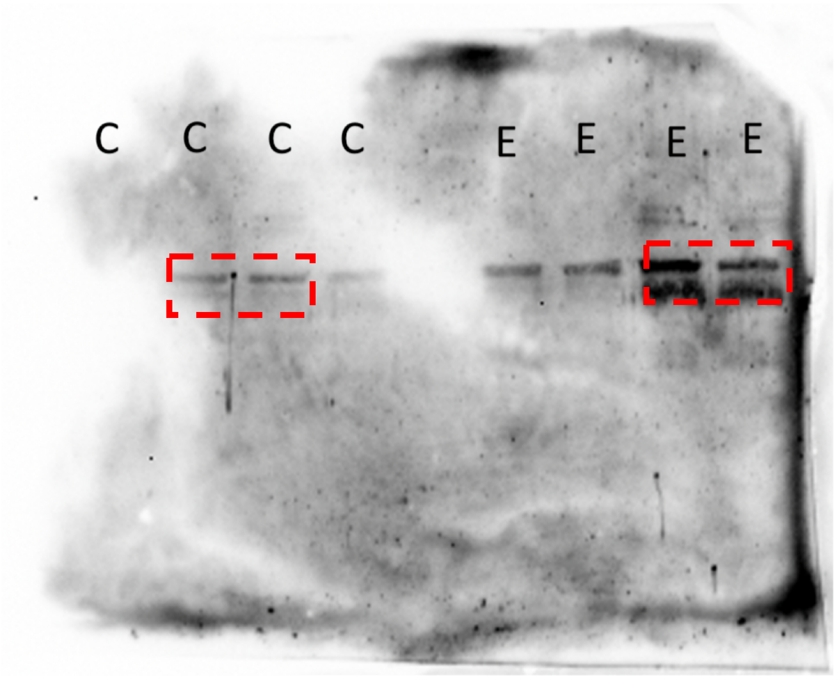

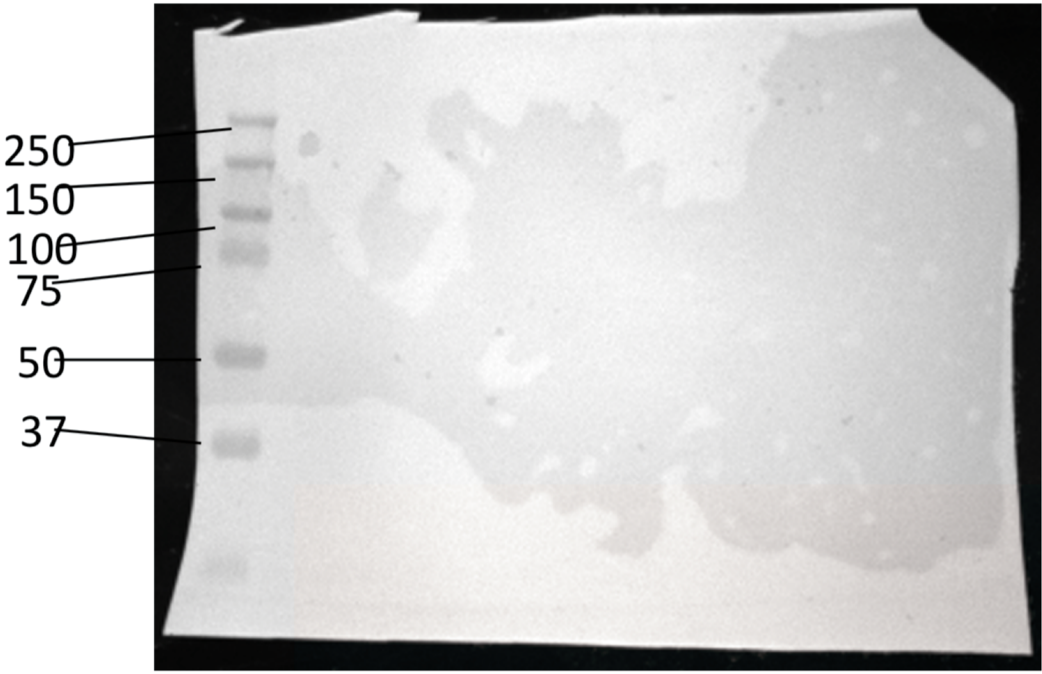

Vinculin 126KDa
YAP (65-78KDa)

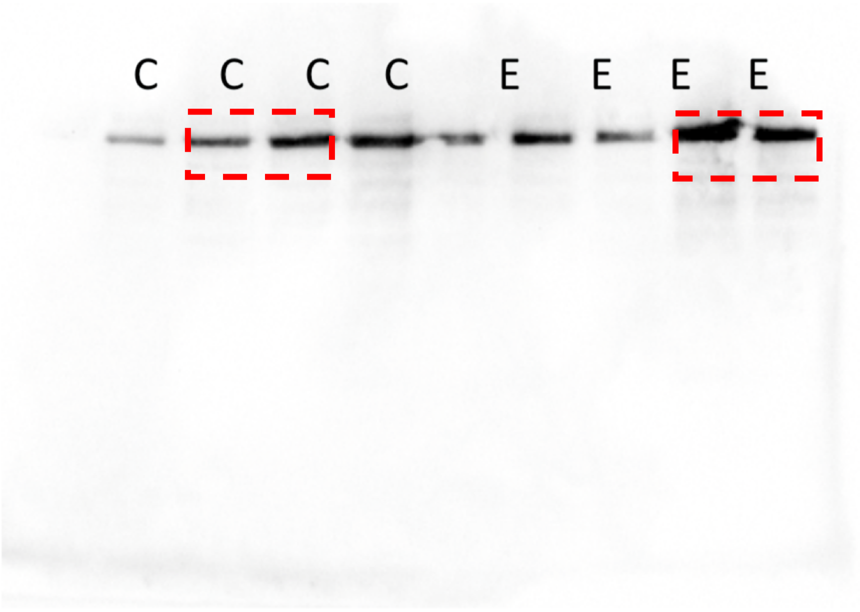

pYAP (65-78 kDa)

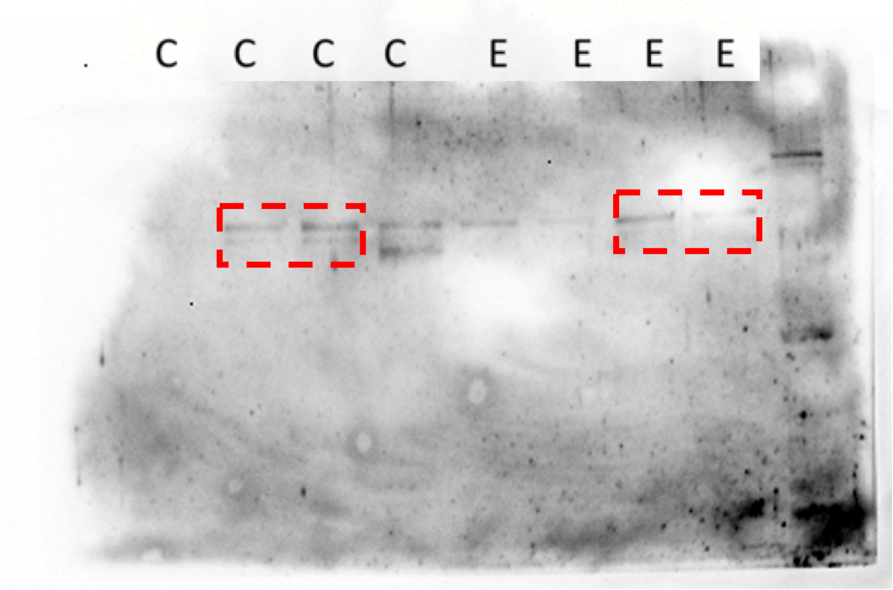

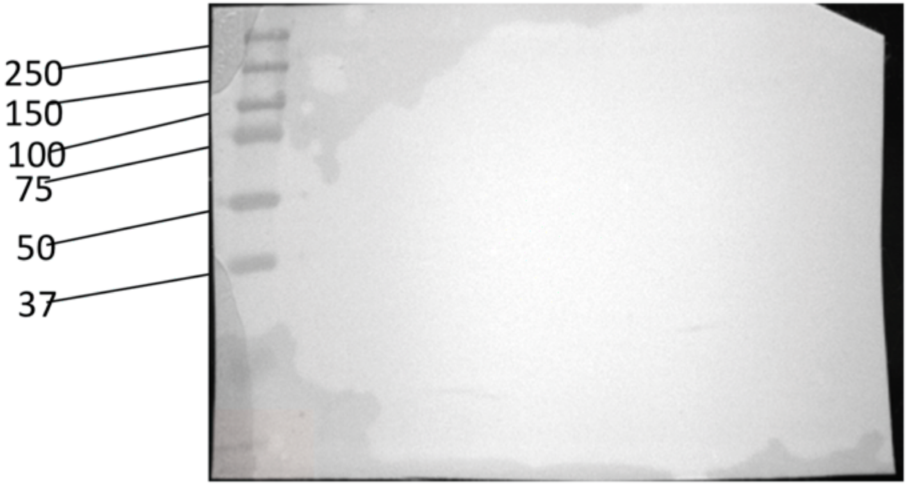

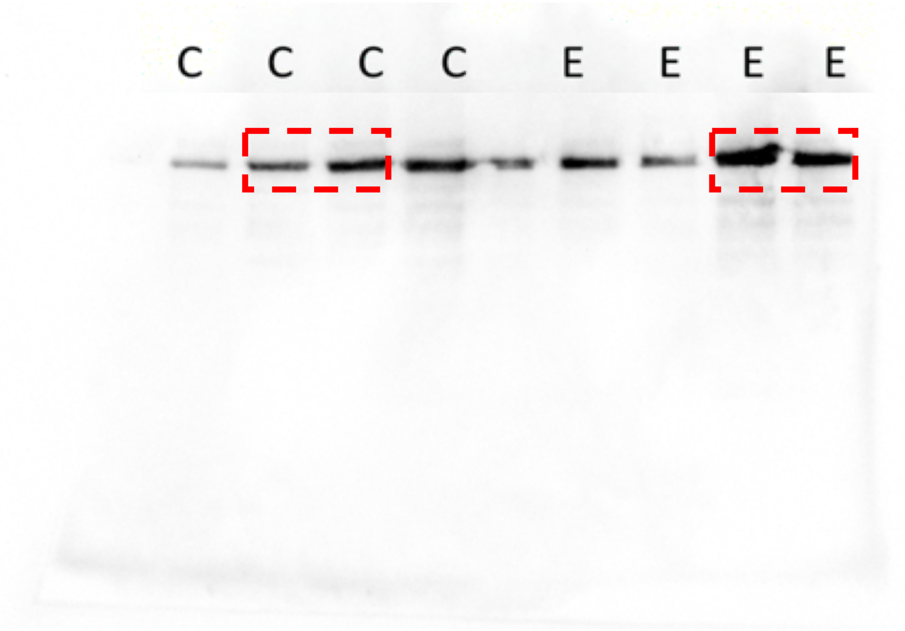

Vinculin (126 kDa)
